# Hyperkalemia in chronic kidney disease patients with and without heart failure: an Italian economic modelling study

**DOI:** 10.1186/s12962-024-00547-y

**Published:** 2024-05-21

**Authors:** Ewa Stawowczyk, Thomas Ward, Ernesto Paoletti, Michele Senni, Antonio Ramirez de Arellano

**Affiliations:** 1 Health Economics and Outcomes Research Ltd, Cardiff, UK; 2 Health Economics Group, College of Medicine and Health, University of Exeter, Exeter, UK; 3 Nephrology, Dialysis and Transplantation, University of Genoa and Policlinico, San Martino Genoa, Italy; 4 Cardiovascular Department and Cardiology Unit, Papa Giovanni XXIII Hospital, Bergamo, Italy; 5 HEOR, CSL Vifor, Glattbrugg, Switzerland

**Keywords:** Cost-effectiveness, Heart failure, Chronic kidney disease, Hyperkalemia, Italy

## Abstract

**Background:**

Hyperkalemia (HK) is frequently present in chronic kidney disease (CKD). Risk factors for HK among CKD patients include comorbidities and renin–angiotensin–aldosterone system inhibitor (RAASi) treatment. Current standard of care (SoC) often necessitates RAASi down-titration or discontinuation, resulting in poorer cardiorenal outcomes, hospitalization and mortality. This study evaluates the cost-effectiveness of patiromer for HK in CKD patients with and without heart failure (HF) in an Italian setting.

**Methods:**

A lifetime Markov cohort model was developed based on OPAL-HK to assess the health economic impact of patiromer therapy in comparison to SoC after accounting for the effects of HK and RAASi use on clinical events. Outcomes included accumulated clinical events, number needed to treat (NNT) and the incremental cost-effectiveness ratio (ICER). Subgroup analysis was conducted in CKD patients with and without HF.

**Results:**

Patiromer was associated with an incremental discounted cost of €4,660 and 0.194 quality adjusted life years (QALYs), yielding an ICER of €24,004. Per 1000 patients, patiromer treatment prevented 275 moderate/severe HK events, 54 major adverse cardiovascular event, 246 RAASi discontinuation and 213 RAASi up-titration/restart. Subgroup analysis showed patiromer was more effective in preventing clinical events in CKD patients with HF compared to those without; QALY gains were greater in CKD patients without HF versus those with HF (0.267 versus 0.092, respectively). Scenario analysis and sensitivity analysis results support base-case conclusions.

**Conclusion:**

Patiromer is associated with QALY gains in CKD patients with and without HF compared to SoC in Italy. Patiromer prevented HK events, enabled RAASi therapy maintenance and reduced cardiovascular event risk.

**Supplementary Information:**

The online version contains supplementary material available at 10.1186/s12962-024-00547-y.

## Introduction

Hyperkalemia (HK) (serum potassium concentration > 5.0 mmol/l) is associated with increased risk of cardiac arrhythmias, muscle weakness/paralysis and mortality [1, 2]. HK occurs due to potassium homeostasis dysfunction and frequently presents in patients with chronic kidney disease (CKD); the presence of other comorbidities (including heart failure (HF), diabetes mellitus and hypertension [3–5]) heightens risk of HK. A retrospective analysis investigating the association between HK prevalence and comorbidities reported that HK events were more frequent in patients with CKD and/or HF versus patients without these comorbidities [6].

An additional risk factor for HK includes renin–angiotensin–aldosterone system inhibitor (RAASi) use, including angiotensin-converting enzyme inhibitors and angiotensin-receptor blockers, the current mainstay treatment options for cardiorenal patients [7]. Optimal RAASi dosing offers renal and cardiovascular protection in patients with CKD and HF, reducing the risk of cardiovascular events, kidney failure and all-cause mortality [5]. Despite the clinical benefits of RAASi, its use in cardiorenal patients is often compromised through discontinuation/down-titration, due to increased HK incidence rates and the requirement to manage potassium levels; resulting in an increased risk of poorer cardiorenal outcomes, hospitalization and mortality [5].

Treatments for HK, such as dietary interventions, loop diuretic therapy, and RAASi discontinuation/down-titration, are not effective long-term. Consequently, HK management in CKD patients with and without HF remains inadequate and further therapeutic development is needed in this clinical setting.

Patiromer, a non-absorbed polymer which binds to potassium within the gastrointestinal tract reducing serum potassium levels, has been approved for the treatment of HK by the European Medicines Agency [8], and reimbursed in five European countries including Italy [9]. Clinical trials, including OPAL-HK, PEARL-HF, AMETHYST-DN, DIAMOND and AMBER, have demonstrated the effectiveness and safety of long-term patiromer use in reducing serum potassium levels in cardiorenal patients receiving RAASi [10–14]. Patiromer therapy could address some of the unmet need in chronic HK management in cardiorenal patients.

In Italy, current HK management is insufficient, with approximately one-third of CKD patients in nephrology clinics remaining with or developing HK annually [15–17]. Although approved in the EU for treating HK in patients with CKD with or without HF [8], economic evaluation of patiromer introduction in an Italian setting is lacking. The objective of this study is to estimate the cost-effectiveness of patiromer for the treatment of HK in patients with CKD with or without HF in Italy, and to explore the influence of HF on outcomes.

## Methods

A previously published lifetime Markov cohort model was adapted to assess the economic impact of patiromer therapy in comparison to standard of care (SoC), in controlling HK in CKD patients with and without HF, from a payer’s perspective [18]. The model was designed to predict the natural history of CKD and HF and quantify the direct medical costs and benefits associated with patiromer use for serum potassium management in patients in Italy. As CKD and HF are chronic progressive diseases associated with increased risk of mortality, a lifetime horizon was modelled in line with technology assessment guidelines, and a monthly cycle length was adopted [19]. A discount rate of 3% was applied to both costs and utilities.

### Model structure

Patients enter the model with either CKD alone or CKD and HF. CKD disease progression was modelled through advanced CKD stages to end-stage renal disease (ESRD), comprising of separate dialysis and transplant health states. In CKD patients with HF, HF disease progression was modelled via transitions between New York Heart Association (NYHA) classifications (I to IV). CKD and HF were modelled independently, with progression through health states in one not impacting progression through health states in the other, except for those exiting the model in the death health state. As a simplifying assumption, patients without HF at model initiation were assumed to not develop HF throughout the modelled time horizon. The distribution of patients among health states at model initiation, alongside baseline age and sex input parameters, were based on the OPAL-HK trial [10] and are presented in Table 1. Disease progression and long-term outcomes, sourced from published literature, are described in Additional file1.

**Table 1:** Health state distributions at baseline

	Mean	SE
**Starting health state distribution**		
Proportion CKD stage 3^a^	55.14%	3.19%
Proportion CKD stage 4^b^	44.86%	3.19%
Proportion CKD stage 5^b^	0.00%	0.00%
Proportion with HF	41.98%	-
Proportion NYHA I	18.63%	3.85%
Proportion NYHA II	64.71%	4.73%
Proportion NYHA III	16.67%	3.69%
Proportion NYHA IV	0.00%	0.00%
**Patient characteristics**		
Age (years)	65.3	0.89
Proportion female	0.46	0.05

**Notes**: ^a^ Included 22/243 patients considered to be in CKD stage 2 at trial entry

^b^ Patients were described only as “stage 4 or worse.”; the proportion of patients pre-RRT in stage 5 is thus unknown and assumed to be 0. **Sources**: OPAL-HK CSR [20] **Abbreviations**: CKD: chronic kidney disease; CSR: clinical study report; HF: heart failure; NYHA: New York Heart Association; RRT: renal replacement therapy

As the simulated cohort progresses through the model, the value of alternative treatments is captured through the occurrence of HK events, changes in RAASi use and treatment discontinuation. The likelihood of clinical events (major adverse cardiac events (MACE), hospitalization and mortality) was predicted and impacted directly by a patient’s health state (e.g., CKD and HF), RAASi use and HK incidence (i.e., potassium level).

### Comparator

Patiromer use was compared against current SoC. Modelling SoC is challenging due to the considerable heterogeneity associated with HK pathogenesis, methods to correct/manage potassium levels (particularly non-pharmacological interventions, and variable levels of adherence to pharmacological methods), and patient responses to such interventions. Accordingly, the broad definition of SoC used in OPAL-HK was adopted and comprised lifestyle interventions for the background maintenance of potassium (e.g., dietary intervention and modification of concomitant medications) and acute management for the correction of potassium. This aligns with current clinical HK management in Italy [17].

### Clinical event incidence

#### HK

HK was classified as a serum potassium level > 5 mmol/l, consistent with definitions used in OPAL-HK and the broader HK literature [21, 22]. Events were further stratified by severity (i.e., of 5-5.5 mmol/l [mild HK], 5.5-6 mmol/l [moderate HK] and > 6 mmol/l [severe HK]). During the first three cycles of the modelled time horizon, incident HK events were based on data from OPAL-HK [21, 23]. For the remaining timeframe, HK annual rates were sourced from European data from Humphrey et al. [24] and applied to the SoC arm [25]. The effect of patiromer use on the rate of HK was obtained from OPAL-HK and incorporated into the model. Table 2 summarizes the applied HK event probabilities. Increased potassium levels negatively impact the occurrence of MACE, hospitalization, and death; the magnitude of these impacts is further described in additional file2.

**Table 2:** Monthly hyperkalemia probability

	Potassium level	Patiromer		SoC		Source
		Mean	SE	Mean	SE	
Month 1	K + > 5 to ≤ 5.5	21.13%	3.32%	21.13%	3.32%	OPAL-HK CSR; distributed across K + categories in line with published data [20]
	K + > 5.5 to ≤ 6	1.66%	1.04%	1.66%	1.04%	
	K + > 6	0.38%	0.50%	0.38%	0.50%	
Month 2 & 3	K + > 5 to ≤ 5.5	14.00%	4.68%	15.00%	4.81%	OPAL-HK CSR [20]
	K + > 5.5 to ≤ 6	6.10%	3.23%	25.22%	5.86%	
	K + > 6	1.40%	1.58%	5.78%	3.15%	
Subsequent months^a^	K + > 5 to ≤ 5.5	0.38%	0.03%	0.80%	0.06%	Humphrey et al [24] OPAL-HK CSR [18]
	K + > 5.5 to ≤ 6	0.08%	0.01%	0.33%	0.03%	
	K + > 6	0.02%	0.00%	0.09%	0.02%	

**Notes**: ^a^SoC probabilities informed by rates of new HK cases observed in Humphrey et al.; patiromer estimates informed by in Humphrey et al. after application of a HR based on OPAL-HK data from months 2 and 3. **Abbreviations**: CSR: clinical study report; HK: hyperkalemia; K+: potassium; RAASi: renin-angiotensin-aldosterone system inhibitor; SE: standard error; SoC: standard of care

#### RAASi use

All patients are assumed to be using a maximal RAASi dose upon entering the model. Patients may reduce their RAASi dose or discontinue RAASi treatment (from any dose) at any point in the model. Continuous RAASi use is known to favorably impact on CKD progression and the incidence of MACE, hospitalization and death, and negatively impact on the incidence of HK. The relationships between these events are further described in additional files 1 and 2. The effect of RAASi dose on clinical events has previously been investigated in CKD and HF patients; for the purposes of defining an optimal versus sub-optimal RAASi dose level, we utilize the definition reported within this study (i.e., < 50% and ≥ 50% of the guideline-recommended RAASi dose for sub-optimal and optimal RAASi dose levels, respectively) [5].

The proportion of patients still on RAASi one month after patiromer and SoC initiation is based on data reported from OPAL-HK [10]. For the patiromer arm, this proportion relates only to those that have achieved response; patients not responding to treatment were assumed to receive RAASi therapy in line with the SoC arm. Rates of RAASi discontinuation and down-titration were taken from OPAL-HK for months 2 and 3 [20]. For the remaining timeframe of the model, RAASi discontinuation and down-titration rates were dependent on potassium levels, based on data from Linde et al., and applied to the SoC arm [5]. The effects of patiromer on RAASi discontinuation/down-titration compared to SoC were obtained from OPAL-HK and incorporated into the model [10]. To reflect transient changes in RAASi regimen, patients were allowed to return to optimal RAASi use, independent of their potassium level, with a monthly probability of 3.51% [5]. Due to a lack of relevant data, patients who down-titrated RAASi use were assumed not to return to maximum use. Rates of RAASi discontinuation and down-titration used in the model are detailed in additional file 1.

#### Treatment discontinuation

Patients initiated on patiromer could discontinue treatment after one month, depending on their response to treatment. In accordance with OPAL-HK, patients responding to patiromer within the first month continued to receive patiromer and were subject to the associated event risks. Those not responding to patiromer discontinued treatment and incurred a risk of events in line with SoC (i.e., assuming no legacy effect of patiromer treatment). In the comparator arm, treatment with SoC could not be discontinued. Beyond the first month, patients receiving patiromer discontinued treatment at a constant monthly probability of 10.33%, or if they reached ESRD, subsequently incurring event risk in line with the SoC arm. Patients in the patiromer arm repeated treatment if their potassium levels were equal to or exceeded 5.5-6 mmol/l in the months following discontinuation.

#### Clinical events

MACE comprised of hospitalizations due to coronary heart disease, HF, ischemic stroke, and peripheral arterial disease as defined in Go et al. [26]. Hospitalization related to all-cause hospitalization. The probability of MACE, hospitalization and mortality, stratified by disease severity, were estimated for a CKD-only and HF-only patient; the higher value was then applied in the CKD cohort with HF. In both cohorts, where all-cause mortality estimates from Italian-specific life Table [27] exceeded mortality estimates based on comorbidities and RAASi use, the greater mortality rate was utilized.

A targeted literature search was carried out to identify studies reporting on the rate of cardiovascular events, hospitalization, and mortality in Italian patients with CKD or HF and HK. The search was carried out in Medline (PubMed) and Google Advanced Search; and was supported with snowballing on relevant articles. Search terms used were grouped into health state concepts (CKD or HF-related), HK-related terms and a search filter for studies conducted in Italy. The best available evidence, identified by relevance of the population, sample size and study robustness, was used to inform model parameters. Event rates are described in additional file 1.

### Costs

Direct medical costs included HK and disease management costs, costs of RAASi use and dose titrations, and one-off event costs of MACE, hospitalization, death and ESRD events (dialysis, transplantation). All costs were reported in 2020/21 Euros.

Healthcare utilization for HK management was primarily derived from Italian guidelines [17] and multicenter prospective observational studies in non-dialysis [16, 25] and dialysis patients [28]. RAASi use was based on OPAL-HK [20] and dose optimization was aligned with technology appraisal guidance from National Institute for Health and Care Excellence (NICE) for sodium zirconium cyclosilicate in treating HK [29]. HK-related hospitalization cost data was taken from Italian diagnostic-related-groups (DRGs) [30]. Drug costs were primarily obtained from the list of class A medicines [31]. Resource utilization and the costing of disease management and clinical events was primarily informed by published literature [28, 32–34]. All costs are summarized in Table 3 and detailed in additional file3.

**Table 3:** Summary of costs input

	Cost (€)		
Parameter	Mean^a^	SE	Source
**Health state costs**			
Annual cost CKD 3 management	2,227.98	146.22	Jommi et al. 2018 [32]; weighted average of CKD 3a and 3b health states
Annual cost CKD 4 management	4,353.68	406.29	Jommi et al. 2018 [32]
Annual cost CKD 5 (pre-RRT) management	5,724.77	625.18	Jommi et al. 2018 [32]
Annual cost of dialysis	33,532.46	476.89	Roggeri et al. 2017 [28]; weighted by dialysis type observed in publication; hospitalization costs subtracted
Dialysis access cost	5,779.33	577.93^b^	Italian Ministry of Health [30]; weighted by dialysis type in Roggeri et al. 2017 [28]
One-off transplant procedure cost	22,197.94	2,219.79^b^	Roggeri et al. 2019 [33]
Annual cost of transplant maintenance	19,077.11	1,797.73	Roggeri et al. 2019 [33]; hospitalization and transplant surgery cost subtracted
**Event costs**			
HK event: K + > 5.5 to ≤ 6.5 mmol/L	73.83	7.38^b^	See Online Resource 3
HK event: K + > 6.5 mmol/L	1,916.11	191.61^b^	See Online Resource 3
MACE	4,464.59	446.46^b^	Corrao et al. 2014 [35]
Hospitalization	3,617.77	361.78	Roggeri et al. 2014 [36]
RAASi discontinuation	84.40	8.44^b^	See Online Resource 3
RAASi down-titration	126.60	12.66^b^	See Online Resource 3
Return to maximum RAASi use	36.92	3.69^b^	See Online Resource 3
**RAASi therapy costs**			
Optimal therapy (Max)	115.58	11.56^b^	See Online Resource 3
Sub-optimal therapy (Sub-max)	57.79	5.78^b^	See Online Resource 3

**Notes:** Costs associated with NYHA health state management were not included and were assumed to be captured within the CKD management (which include general health care expenditure), hospitalization and MACE costs)

^a^Where necessary, costs were inflated to 2020/21 values using the Italian consumer price index reported by The World Bank [50]

^b^SE values assumed 10% of the mean. **Abbreviations**: CKD: chronic kidney disease; HK: hyperkalemia; K+: potassium; MACE: major adverse cardiac event; NHYA: New York Heart Association; RAASi: renin–angiotensin–aldosterone system inhibitor; RRT: renal replacement therapy

### Health utilities

Health utilities (and disutilities) applied to modelled health states (and events) are presented in additional file 4 [37–42]. Utility estimates measured with the EQ-5D were broadly informed by a recent NICE technology appraisal [29]. A targeted literature search carried out in Medline (PubMed) and Google Advanced Search did not identify any Italian-specific studies reporting on quality of life that could be deemed more appropriate than those informed by the NICE technology appraisal.

### Analysis

The costs, life years and quality-adjusted life years (QALYs) accumulated by each treatment arm were reported. Comparisons between treatments were made utilizing the incremental cost-effectiveness ratio (ICER). The number of clinical events occurring throughout the modelled time horizon and the number of patients needed to treat (NNT) with patiromer to prevent one additional case of HK, MACE, hospitalization and RAASi discontinuation/down-titration were calculated. In sub-group analysis, these calculations were repeated in CKD patients with and without HF, where all other model input parameters were assumed to remain the same as in the base case analysis.

In scenario analysis, the effect of RAASi use on clinical events (i.e., MACE and hospitalization) was incorporated into the model using an alternative definition of optimal RAASi use reported in Italian-specific studies [43, 44]. Within these data sources, persons with > 80% proportion of days covered (PDC) with RAASi were defined as adherent, and subsequently informed risk in the optimal RAASi use group in the model. Conversely, those with ≤ 80% PDC were assumed to represent those not receiving RAASi in the model. PDC is one of the most reliable methods for measuring medication adherence in chronic therapies; a threshold of 80% is indicative of achieving maximum clinical benefit [45].

Italian-specific data were also incorporated into scenario analysis, including alternative assumptions around the baseline distribution of patients among health states, the proportion of patients with HF and baseline demographic and clinical risk factors. Further details on the data applied in scenario analyses are presented in additional file5.

### Sensitivity analysis

One-way sensitivity analysis was undertaken to assess the impact of individual model parameters on the ICER; the most influential and uncertain input parameters were incorporated in the analysis. The discount rate was varied between 0% and 5%, as required by Italian guidelines [19]. Probabilistic sensitivity analysis was undertaken, with parameter values sampled independently across 5,000 model iterations. Patient characteristics were sampled using a normal distribution; probabilities and health utilities were sampled using a beta distribution; and costs, hazard ratios and odds ratios were sampled using a gamma distribution.

## Results

### Base-case analysis

Base-case results are presented in Table 4. Treatment with patiromer was associated with an additional discounted €4,660 and 0.194 QALYs, yielding an ICER of €24,004. Compared to SoC, life expectancy was extended by 0.256 life years. Differences in costs were primarily driven by patiromer treatment and CKD/ESRD disease management. Compared to SoC, patiromer use was associated with higher CKD management costs and renal replacement therapy (RRT) costs, with patients spending more time in pre-dialysis disease stages (due to reduced CKD progression) and observing greater life expectancy. Costs associated with HK events and MACE were also reduced in the patiromer arm. Furthermore, the distribution of costs relating to RAASi use and titration indicates more persons remain on RAASi treatment when receiving patiromer.

**Table 4:** Cost-effectiveness results

	Base-case analysis				CKD subgroup			CKD with HF subgroup		
	Treatment		Control	Δ	Treatment	Control	Δ	Treatment	Control	Δ
**Discounted results**										
Total costs (€)	124,735	120,075		4,660	138,989	133,020	5,969	105,378	102,540	2,838
Treatment (€)	3,115	-		3,115	3,280	-	3,280	2,892	-	2,892
HK (€)	814	930		-116	845	960	-115	772	890	-118
CKD (€)	27,078	26,244		834	29,893	28,821	1,072	23,291	22,787	503
RRT (€)	75,387	74,567		820	87,308	85,815	1,493	59,178	59,306	-128
MACE (€)	9,529	9,675		-146	9,801	9,815	-14	9,144	9,472	-328
Hospitalization (€)	7,892	7,802		90	6,841	6,663	178	9,319	9,350	-31
RAASi usage (€)	569	477		92	635	534	101	481	401	80
RAASi titration (€)	349	379		-30	386	413	-27	300	334	-34
Total QALYs	6.624	6.430		0.194	8.016	7.749	0.267	4.747	4.655	0.092
Total LYs	9.525	9.268		0.256	10.664	10.329	0.336	7.984	7.839	0.145
ICER (€/QALY)	-	-		24,004	-	-	22,324	-	-	30,875
**Undiscounted results**										
Total costs	170,809	164,612		6,197	194,040	185,935	8,083	139,340	135,773	3,567
Total QALYs	8.311	8.055		0.257	10.151	9.795	0.356	5.839	5.720	0.118
Total LYs	12.055	11.709		0.346	13.711	13.255	0.457	9.821	9.631	0.190
ICER (€/QALY)	-	-		24,155	-	-	22,729	-	-	30,109

**Abbreviations**: CKD: chronic kidney disease; HF: heart failure; HK: hyperkalemia; ICER: incremental cost-effectiveness ratio; K+: serum potassium level; LY: life years; MACE: major adverse cardiac event; QALY: quality-adjusted life years; RAASi: renin–angiotensin–aldosterone system inhibitor; RRT: renal replacement therapy

**Fig. 1:**
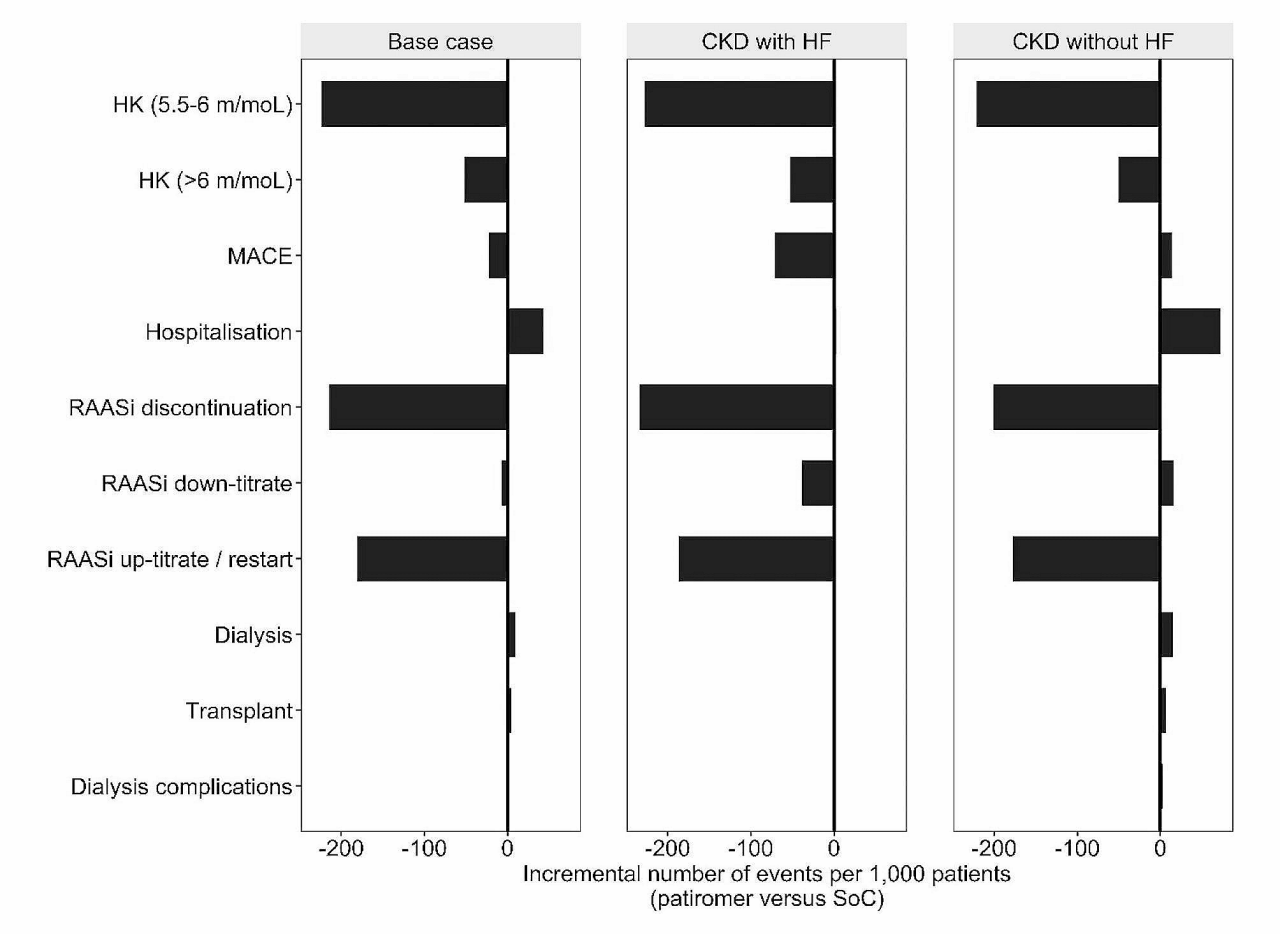
Incremental number of lifetime clinical events per 1,000 patients (patiromer versus SoC). Abbreviations: CKD: chronic kidney disease; HF: heart failure; HK: hyperkalemia; MACE: major adverse cardiac event; RAASi: renin?angiotensin?aldosterone system inhibitor; SoC: standard of care

Patients received patiromer treatment for an average of 10.4 months. The number of incremental events accrued by patients over the modelled time horizon are illustrated in Fig. 1. Per 1,000 patients, 224 moderate HK (5.5-6 mmol/l) events and 51 severe HK (≥ 6 mmol/l) events were avoided with patiromer use. The NNT with patiromer to avoid an additional case of HK was 4.5 and 19.4 for moderate and severe HK, respectively. Compared to SoC, there were fewer cases of MACE and RAASi discontinuation and subsequent up-titration with patiromer use. The NNT with patiromer to prevent one additional case of MACE and RAASi discontinuation was 45.6 and 4.7 patients, respectively. Event counts of RAASi down-titration, hospitalization, dialysis and transplant were similar between arms.

### Subgroup analysis

Patiromer was associated with incremental costs and survival and QALY gains in patients with CKD and HF and in patients with CKD alone. Discounted incremental costs were €2,838 in those with CKD and HF and €5,969 in those with CKD alone, while discounted QALY gains were 0.092 and 0.267, respectively (Table 4). Differences in outcomes were predominantly driven by the greater rate of mortality amongst patients with comorbid HF. Over the modelled time horizon, patients with CKD and HF in the patiromer arm observed an average life expectancy of 9.8 years (SoC: 9.6 years), while those without HF observed an average life expectancy of 13.7 years (SoC: 13.3 years).

Whilst the total number of clinical events was lower in the CKD and HF subgroup compared to CKD alone (Additional file6), the number of HK events (≥ 5.5 mmol/l) avoided with patiromer, NNT and associated costs were similar across sub-groups and base-case results. The need to discontinue RAASi therapy was substantially reduced with patiromer in both subgroups, although this reduction was more pronounced in the CKD and HF subgroup. Across subgroups, 4–5 patients would need to be treated with patiromer to avoid one additional case of RAASi discontinuation. The number of events avoided through patiromer use for each subgroup are presented in Fig. 1. Cost savings were realized through reductions in the number of HK events, MACE events (although only in the cohort with HF) and RAASi discontinuation episodes in both subgroups. Patiromer was associated with 272 fewer moderate and severe HK events, 14 additional MACE events and 201 fewer RAASi discontinuation episodes in the CKD without HF subgroup per 1,000 patients. For the CKD with HF subgroup, the number of events avoided were 280 moderate and severe HK events, 71 MACE events and 234 RAASi discontinuation episodes, per 1,000 patients. This equated to a NNT of 3.6–3.7 to avoid one HK event, 14 to avoid one MACE event (in the subgroup with HF), and 4.3-5.0 to avoid one RAASi discontinuation episode. The extension of life associated with patiromer in patients with CKD without HF resulted in an additional 73 hospitalizations and 22 RRT events per 1,000 patients, and an additional 3 hospitalization and 2 RRT events per 1,000 patients with CKD with HF.

### Sensitivity analysis

Changing the discount rate applied to costs and benefits, respectively, resulted in ICER estimates varying from €20,618 to €31,924 and €18,163 to €28,194 under base case assumptions. In wider-ranging one-way sensitivity analyses, the most influential parameters were the model discount rates, the patiromer discontinuation rate, the cost of patiromer, the influence of RAASi use on CKD disease progression rates and RAASi discontinuation and baseline patient age. The sensitivity analysis tornado plot is shown in Additional file7.

**Fig. 2:**
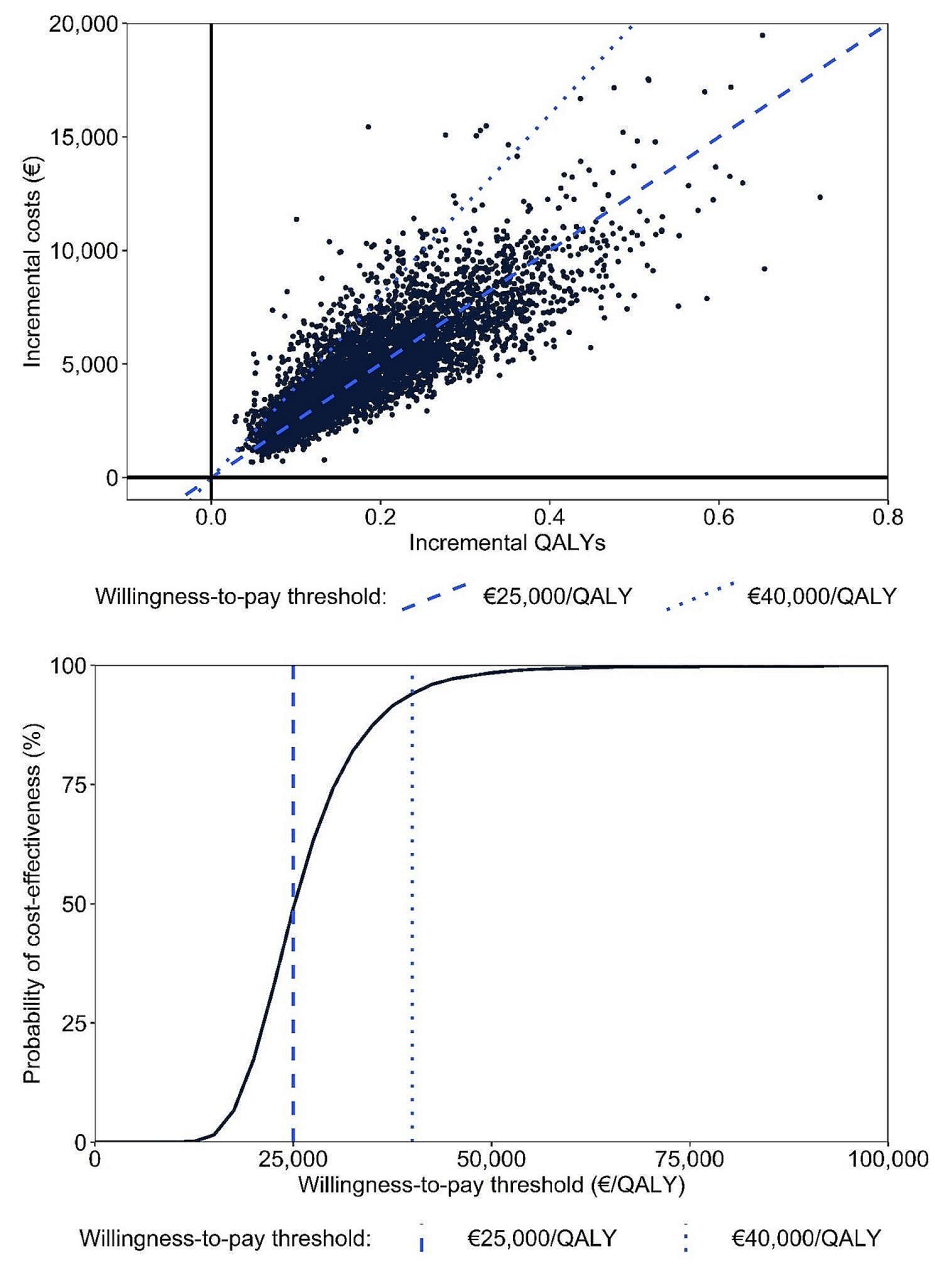
Results of probabilistic sensitivity analysis. Upper figure shows the scatterplot of incremental costs and QALYs. Lower figure displays the cost-effectiveness acceptability curve. **Abbreviations**: QALY: quality-adjusted life years

Results of probabilistic sensitivity analyses, presented in Fig. 2, support the deterministic base case analysis. Under base case assumptions, the discounted incremental cost and QALY gains were €4,887 and 0.191, respectively, resulting in an ICER of €25,553. Patiromer was estimated to have a 49.1% and 94.0% probability of cost-effectiveness, compared to SoC, at willingness-to-pay thresholds of €25,000 and €40,000, respectively.

### Scenario analysis

Utilizing alternative sources of RAASi influence on transient events (i.e., different definitions of optimal RAASi use, approximated through estimates of adherence) increased discounted incremental cost and QALY outcomes to €5,326 and 0.216, respectively, resulting in an ICER of €24,693. The average increase in life expectancy associated with patiromer use was estimated to be 0.407 years.

The introduction of alternative Italian-specific baseline patient data to the model increased discounted incremental costs and QALY outcomes to €5,244 and 0.191, respectively, yielding an ICER of €27,506. Patiromer use was associated with an average life expectancy gain of 0.343 years.

## Discussion

Our results demonstrate that HK treatment with patiromer in CKD patients with and without HF increases quality of life outcomes compared to SoC in Italy. From a healthcare payer’s perspective, the model estimated that the introduction of patiromer was associated with an incremental discounted lifetime benefit of 0.194 QALYs, with an incremental discounted cost of €4,660, yielding an ICER of €24,004 per QALY in comparison to SoC. Despite a small incremental cost associated with patiromer use, attributable to both initial patiromer treatment and increased CKD and ESRD management costs, cost-offsets were made through reductions in MACE and HK costs. Patiromer was able to prevent future HK events; enabling patients to maintain RAASi therapy and reduce their risk of cardiovascular events. Subsequently, patients progressed at a slower rate to RRT and had more time to accrue CKD-related healthcare costs. In our subgroup analyses, the beneficial effects of patiromer are observed in both CKD patients with and without HF, and are most pronounced in patients with CKD alone, due to their longer life expectancy and longer time available to accrue the benefit of patiromer use. Together with scenario and sensitivity analyses, our results highlight the importance of continuous RAASi therapy in CKD patients both with and without HF.

In Italy, HK presents a significant clinical and economic burden, with increased HK prevalence in CKD and HF populations associated with worsening outcomes and lower RAASi use [15–17, 44, 46]. A community database study of 12 million inhabitants investigated the prevalence of HK in the general Italian population; although HK prevalence was low among the general population (0.035%), more than half of those affected were hospitalized over a one year period, with tripling costs to the Italian healthcare system [46]. This study also examined the burden and prevalence of HK in patients with HF, using data from the Italian Network on Heart Failure (IN-HF) registry [46]. Authors reported that HK was frequent in patients with CKD and/or HF and was associated with decreased RAASi use compared to patients with normokalemia. Furthermore, an Italian observational study assessed outcomes and determinants of HK in 2,446 CKD patients across 46 nephrology clinics, and found that HK (mild to moderate) was prevalent amongst 37% of CKD patients with an associated risk of ESRD progression [16]. These studies highlight the unmet need for better treatment options for HK in CKD patients with and without HF in Italy.

The clinical benefits of reducing potassium levels are paramount in allowing CKD patients with and without HF to maintain RAASi therapy. Enabling optimal dosage of RAASi therapy is associated with improvement of cardiovascular and renal outcomes, including attenuation of disease progression [16, 43] and improved survival [16, 43, 44, 47] in CKD and/or HF patients.

Whilst RAASi enablement is important for reducing the rate of events in HF, a patient’s ability to realize this gain is limited due to the significantly reduced life expectancy in CKD patients with HF. In the elderly population, whom are most at risk of developing comorbid CKD and HF, there is a significant increased risk associated with cardiovascular-related hospitalizations and mortality [48]. Since there were greater reductions in hospitalizations in the CKD with HF group compared to CKD alone, in this instance, a treatment which enables optimal use of RAASi therapy would greatly reduce hospitalization. Patiromer therapy was associated with an incremental gain of 0.256 LY in the base-case analysis and 0.145 LY for the CKD with HF group. These are clinically meaningful increases (2.8% and 1.9%) over their respective control arms; for the CKD with HF cohort, this is approximately equivalent to 53 days.

Our cost-effectiveness analysis was based on OPAL-HK, which had its limitations. Long-term outcomes for patiromer are not yet available, hence extrapolation of treatment outcomes, measured over 3 months in OPAL-HK, were required to approximate the longer-term impact of patiromer, introducing uncertainty to estimates of long-term cost-effectiveness. The design of the trial (i.e., a two-part, single-blind study) may have contributed to an underestimation in the relative benefit of patiromer compared to SoC, as patients in the SoC arm initially received patiromer, which was then withdrawn over subsequent months. The RAASi discontinuation algorithm utilized in OPAL-HK may not accurately represent discontinuation in all healthcare settings and may have overestimated the proportion of patients discontinuing RAASi therapy in the SoC arm. Both limitations are considered in one-way sensitivity analyses. OPAL-HK enrolled patients from different countries, with only a small fraction from Italy, hence there may be discrepancies between trial and real-world Italian outcomes.

Until the publication of the DIAMOND study [49], studies evaluating the association between HK, RAASi and clinical outcomes in CKD patients with and without HF have been limited. Hence, a targeted literature search attempted to identify the best available evidence to inform disease progression and clinical outcomes. Inconsistencies in HK definitions between studies limited the utilization of Italian-specific data in the model, although the UK studies used to inform rates are believed to be representative. A further data limitation concerns the use of the Framingham risk equation for HF mortality, which while utilized widely in contemporary health economic evaluations, is derived from an old data source and is US-specific, potentially overestimating mortality risk. It is likely that this results in an underestimation of the true lifetime benefit of patiromer. Finally, due to limited data availability, hospitalization, MACE, and death were modelled independently; the ability to more accurately relate these clinical events to each would likely improve the real-world validity of the model. Despite these limitations, the methodology applied is rigorous and the data sources represent appropriate approximations of real-world practice and outcomes; this study can be viewed as an indicative first step towards the health economic evaluation of patiromer for the treatment of HK in patients with CKD with and without HF, a complicated and multi-faceted disease area.

To conclude, our results indicate the health economic value of patiromer for the treatment of HK in CKD patients with and without HF in an Italian setting, with cost savings and QALY gains. Improving HK management in Italy by expanding treatment options has the potential to greatly benefit CKD patients with or without HF by enabling optimal RAASi therapy and reducing risk of hospitalization and mortality.

### Electronic supplementary material

Below is the link to the electronic supplementary material.

**Additional file 1**: Disease progression. Provides details of the disease progression data utilized in the model

**Additional file 2**: Disease progression and events. Provides details of the disease progression and events utilized in the model

**Additional file 3**: Costs. Provides detail of cost data stylized in the model. All costs are presented in 2021 EUR

**Additional file 4**: Utility and disutility. Provides details of utility and disutility input parameters utilized in the model

**Additional file 5**: Scenario analysis. Provides additional data utilized in scenario analyses

**Additional file 6**: Subgroup analysis. Provides details of cumulative clinical events in patients with CKD and HK versus CKD alone

**Additional file 7**: Sensitivity analysis tornado. Provides results of deterministic sensitivity analysis

## Data Availability

The data that support the findings of this study are available from CSL Vifor but restrictions apply to the availability of these data, which were used under license for the current study, and so are not publicly available. Data are however available from the authors upon reasonable request and with permission of CSL Vifor.
