# Supplementary material for: Hyperkalemia in chronic kidney disease patients with and without heart failure: an Italian economic modelling study
Source: Cost Eff Resour Alloc. 2024 May 21;22:42. doi: 10.1186/s12962-024-00547-y (PMC11106859; doi:10.1186/s12962-024-00547-y)
Supplement: Supplementary file 1 — Additional file 1: Disease progression. Provides details of the disease progression data utilized in the model [file 12962_2024_547_MOESM1_ESM.pdf]

Additional file 1

This appendix provides details of disease progression data utilized in the model.

1. Baseline patient characteristics

Additional patient characteristics beyond those specified in the main manuscript document are required to estimate the likelihood of death in patients with HF. **Table 1** summarizes these additional patient characteristics. All patient characteristics described in this table remain constant over the course of the simulation (i.e., equal to the specified baseline value).

Table 1: Baseline characteristics available to describe the modelled patient population

Baseline patient characteristic	Mean	SE	Source
Baseline age (years)	65.30	0.89	OPAL-HK CSR[1]
Proportion of cohort female	0.46	0.05	
Ejection fraction (mL)	21.00	0.18	PRAISE[2]
Ischemic etiology (%)	64.00%	1.43%	
Systolic blood pressure (mmHg)	144.58	14.46	Approximated from OPAL-HK CSR; SE taken as 10% of mean[1]
Total cholesterol (mg/dL)	187.60	3.71	OPAL-HK CSR[1]
Hemoglobin (g/dL)	13.90	0.05	PRAISE[2]
Percent lymphocytes (%)	26.00%	1.31%	
Sodium (mEq/L)	139.60	0.27	OPAL-HK CSR[1]
Uric acid (mg/dL)	8.90	0.08	PRAISE[2]
K+ sparing diuretics	56.29%	4.04%	Assumption based on OPAL-HK CSR[1]
Beta blocker	54.30%	4.05%	OPAL-HK CSR[1]
Statin	8.00%	0.81%	PRAISE[2]
Allopurinol	10.00%	0.89%	
ICD	0.00%	0.00%	
BICD	0.00%	0.00%	
Proportion of RAASi users on ACE inhibitors	67.55%	3.81%	OPAL-HK CSR[1]
Proportion of RAASi users on ARB	39.07%	3.97%	
Diuretic dose (mg/kg)	1.45	0.04	PRAISE[2]

ACE: angiotensin converting enzyme; ARB: angiotensin II receptor blocker; BICD: biventricular implantable cardioverter defibrillator, BMI: body mass index; ICD: implantable cardioverter defibrillator; RAASi: renin-angiotensin-aldosterone system inhibitor; SE: standard error.
*Note in the OPAL-HK CSR, patients were described only as "stage 4 or worse."[1] The proportion of patient's pre-RRT in stage 5 is thus unknown and here taken as 0.

2. Natural history evolution of risk factors

Over the course of the simulation, changes from baseline may be modelled for the following risk factors:

- Age
- RAASi use
- NYHA class

- CKD stage

Age is incremented in line with the model clock (i.e., progression of simulated time). The natural history evolution of CKD and HF are modelled via health state transition probabilities. Changes in RAASi use are modelled based on cyclic probabilities of discontinuation and down-titration stratified by HK event status. Re-initiation of RAASi use may also be modelled.

3. Natural history of CKD and ESRD progression

The progression through CKD stages and ESRD is modelled according to health state transition probabilities. Progression through CKD stages and ESRD is tracked based on the transition probabilities reported in **Table 2**. The rate of progression to ESRD is demonstrably related to RAASi use and therefore, the ability to apply a hazard ratio to the progression rates from CKD stage 3 to CKD stage 4 and from CKD stage 4 to CKD stage 5 (ESRD), for those receiving RAASi therapy is made available[3].

Following the initiation of RRT, simulated patients are modelled within an RRT module. Within the RRT module, RRT is broken down into dialysis and transplant health states. Changes in modality of care and the incidence of dialysis-related complications and mortality are controlled via the parameters detailed in **Table 2**.

Calculations used to derive CKD natural history inputs are summarized in **Table 3** to **Table 8**. Additional inputs relating to the calculation of age-dependent ESRD inputs used in RAASi scenario analyses are detailed in Error! Reference source not found. and Error! Reference source not found.**.**

Table 2: Model inputs controlling initiation and management of RRT (RRT module)

Parameter	Mean	SE	Source
**Monthly transition probability**			
CKD stage 3 to CKD stage 4	2.446%	1.127%	Cozzolino et al.[4]
CKD stage 4 to CKD stage 5	1.409%	0.819%	
CKD stage 5 to dialysis	3.944%	2.190%	Cozzolino et al.[4]; Chance of stage 5 progression distributed in line with proportion receiving dialysis or transplant first in Italy based on 2019 RIDT report[5]
CKD stage 5 to transplant	0.056%	0.266%	
Dialysis to Transplant	1.205%	0.120%*	Italian National Transplant Centre report[6]; To approximate the proportion of patients on the transplant waiting list we take the number of new enrolments in 2019 (3,039) and divide by the incidence of dialysis cases in Italy from the 2019 RIDT report[5]
Dialysis to Death	1.473%	0.147%*	2019 RIDT report[5]; one-year mortality estimates weighted by dialysis modality
Transplant to Dialysis (graft failure)	0.207%	0.028%	Italian National Transplant Centre report{Italian National Transplant Centre (Centro
Transplant to Death	0.137%	0.023%	

			Nazionale Trapianti); five year graft survival (censored for death) and mortality estimates
**CKD progression odds ratio**			
On RAASi: CKD stage 3 to CKD stage 4	0.620	0.059	Xie et al.[3]
On RAASi: CKD stage 4 to CKD stage 5 (ESRD)	0.620	0.059	Xie et al.[3]
eGFR: estimated glomerular filtration rate; HF: heart failure; RAASi: renin–angiotensin–aldosterone system inhibitor; SE: standard error<br>*SE assumed 10% of mean			

Table 3: Proportion in receipt of peritoneal dialysis or hemodialysis

Modality	Proportion	Source
Hemodialysis	0.55	2019 RIDT report[5]
Peritoneal dialysis	0.08	
**Distribution after adjustment**		
Hemodialysis	0.873	Calculated (proportions inflated to sum to 100%)
Peritoneal dialysis	0.127	

Table 4: Annual probability of dialysis complications

Parameter	HD	PD	Source
**Number of events**			
MRSA	80	1	Renal Registry (Table 6.8)[7]
MSSA	1,271	29	
C. difficile	498	56	
E. coli	924	67	
Total	2,773	153	
**Infection rate per 100 patient years**			
MRSA	0.17	0.01	Renal Registry (Table 5.8)[7]
MSSA	2.72	0.43	
C. difficile	1.06	0.83	
E. coli	1.96	0.99	
Sum of rates	5.91	2.26	Calculated
**Distribution**			
Proportion on modality	0.873	0.127	See **Table 3**
**Weighting**			
Weighted rate per 100 patient years	5.447		Calculated based on weighted distribution across modalities
Weighted monthly probability (%)	0.453% (0.045%)*		

C. difficile: *Clostridium difficile*; *E. coli*: *Escherichia coli*; HD: hemodialysis; MRSA: methicillin-resistant Staphylococcus aureus; MSSA: methicillin-sensitive Staphylococcus aureus
*SE assumed 10% of mean

Table 5: Monthly probability of dialysis death

Parameter	Estimate	Source
**One-year probability of mortality**		
Hemodialysis [A]	17.0%	2019 RIDT report[5]
Peritoneal dialysis [B]	11.4%	
**Weighted monthly probability**	**1.473%**	Calculated*

HD: hemodialysis; PD peritoneal dialysis
*Used to calculate the monthly probability of dialysis death using the following formula: Monthly probability of dialysis death = 1 - EXP((HD proportion × ln(1 – [A]) + PD proportion × ln(1 – [B])) / 12)

Table 6: Monthly probability of progression from CKD stage 5

Parameter	Estimate	Source
Three-year probability of CKD progression [A]	77.0%	Cozzolino et al.[4]
Likelihood of receiving transplant as first modality [B]	1.4%	2019 RIDT report[5]
**Weighted monthly probability**		
CKD stage 5 to dialysis*	3.944%	Calculated
CKD stage 5 to transplant**	0.056%	

HD: hemodialysis; PD peritoneal dialysis
*Used to calculate the monthly probability of CKD stage 5 progression to dialysis using the following formula: Monthly probability of CKD stage 5 to dialysis = (1 - EXP(ln(1 – [A]) / 36)) × (1 – [B])
**Used to calculate the monthly probability of CKD stage 5 progression to transplant using the following formula: Monthly probability of CKD stage 5 to transplant = (1 - EXP(ln(1 – [A]) / 36)) × [B]

Table 7: Monthly probability of transplant from dialysis

Parameter	Estimate	Source
Annual transplant incidence per million people [A]	162	2019 RIDT report[5]
Total Italian population size [B]	45,607,293	
Annual number of new enrolments on transplant waiting list [C]	3,039	Italian National Transplant Centre report[6]
Annual probability of transplant when on transplant waiting list [D]	30.0%	
**Monthly probability of transplant from dialysis**	**1.205%**	Calculated*

CKD: chronic kidney disease
*Calculated as (1-EXP(ln(1-[D])/12)) × [C] / ([A] × [B] / 1,000,000)

Table 8: Annual probability of graft failure and transplant survival

Parameter	Estimate	Source
Five-year probability of graft survival [A]	88.3%	Italian National Transplant Centre report[6]
Five-year probability of patient survival [B]	92.1%	
**Monthly probability**		
Graft survival*	0.207%	Calculated
Patient survival**	0.137%	

*Used to calculate the monthly probability of graft survival using the following formula: Monthly probability of graft survival = 1 - EXP(ln([A]) / 60)
**Used to calculate the monthly probability of patient survival using the following formula: Monthly probability of patient survival = 1 - EXP(ln([B]) / 60)

4. Natural history of HF progression

Changes in NYHA classification are controlled using monthly probabilities. The model enables the application of different probabilities according to RAASi use (any versus none); however, these are assumed to be equal in the absence of appropriate data (**Table 9**).

Table 9: Monthly probabilities of changes in NYHA classification, with and without RAASi (any use)

		To			
		**NHYA I**	**NYHA II**	**NYHA III**	**NYHA IV**
**From**	**NHYA I**	0.7956	0.1245	0.0738	0.0061
	**NYHA II**	0.0710	0.8448	0.0765	0.0077
	**NYHA III**	0.0047	0.0893	0.8845	0.0216
	**NYHA IV**	0.0000	0.1064	0.1064	0.7872

NYHA: New York Heart Association.
Source: Yao et al.[8]
Source publication did not report sufficient data to support sampling of parameters from dirichlet distribution; thus, parameters are sampled independently with SE derived from the number of patients informing the transition probabilities (n=380)

5. RAASi usage inputs

The calculations for RAASi discontinuation, down-titration and up-titration are presented in **Table 10** and **Table 11**.

Table 10: RAASi discontinuation and down-titration (month 2-3)

	**SoC**			**Patiromer**			**Source**
	**No change**	**RAASi dose reduced by 50%**	**RAASi stopped**	**No change**	**RAASi dose reduced by 50%**	**RAASi stopped**	
**Number of RAASi dose reductions and discontinuations**							
Week 1	44	4	1	53	0	0	OPAL-HK CSR[1]
Week 2	37	6	4	53	0	0	
Week 3	37	3	2	50	0	1	
Week 4	26	8	5	49	0	0	
Week 5	22	3	7	46	0	1	
Week 6	24	0	3	46	0	0	
Week 7	20	2	1	43	0	1	
Week 8	18	1	3	43	0	0	
Total	228	27	26	383	0	3	
Weekly transition probability*	81.14%	9.61%	9.25%	99.22%	0.00%	0.78%	Calculated
Monthly transition probability	-	35.55%	34.44%	-	0.00%	3.34%	
RAASi: renin-angiotensin-aldosterone system inhibitor; SE: standard error; SoC: standard of care<br>*Calculated as number of transitions observed divide by total number of transitions							

Table 11: RAASi discontinuation and down-titration (month 4+)

	Potassium threshold: 5.0 mmol/L		Potassium threshold: 5.5 mmol/L		Potassium threshold: 6.0 mmol/L		Source
	**Below threshold**	**Above threshold**	**Below threshold**	**Above threshold**	**Below threshold**	**Above threshold**	
**Dose modification of renin–angiotensin–aldosterone system inhibitor prescriptions ending within 7 days of a serum potassium (K+) measurement, stratified by serum K+ threshold for CKD patients.**							
Probability of discontinuation	2.6% (2.6-2.7%)	3.7% (3.5-3.9%)	2.7% (2.6-2.8%)	5.6% (5.1-6.0%)	2.8% (2.7-2.9%)	10.0% (8.7-11.3%)	Linde et al.[9]
Probability of down-titration	1.8% (1.8-1.9%)	3.5% (3.3-3.7%)	2.0% (1.9-2.1%)	6.0% (5.5-6.4%)	2.1% (2.1-2.2%	8.9% (7.6-10.1%)	
Total number of patients	132,840	39,181	161,795	10,226	170,046	1,975	
**Monthly probability of discontinuation and down-titration (K+ <5.0 mmol/L); Mean (SE)**							
Discontinuation	2.600% (0.026%)						Linde et al.[9]
Down titration	1.800% (0.026%)						
**Monthly probability of discontinuation and down-titration (K+ 5.0-5.5 mmol/L); Mean (SE)**							
Discontinuation	(39181 * 0.037 – 10226 * 0.056) / (39181 - 10226) = 3.029% (0.102%)						Calculation (Linde et al.[9])
Down titration	(39181 * 0.035 – 10226 * 0.06) / (39181 - 10226) = 2.617 (0.102%)						
**Monthly probability of discontinuation and down-titration (K+ 5.5-6.0 mmol/L); Mean (SE)**							
Discontinuation	(10226 * 0.056 – 1975 * 0.1) / (10226 - 1975) = 4.547% (0.230%)						Calculation (Linde et al.[9])
Down titration	(10226 * 0.06 – 1975 * 0.089) / (10226 - 1975) = 5.306% (0.230%)						
**Monthly probability of discontinuation and down-titration (K+ >6.0 mmol/L); Mean (SE)**							
Discontinuation	10.000% (0.663%)						Linde et al.[9]
Down titration	8.900% (0.638%)						
CKD: chronic kidney disease; SE: standard error<br>Standard errors were calculated by taking the range from the confidence interval and dividing through by 2 and then dividing through by 1.96							

6. Incidence of events

Table 12 summarizes the approach taken to model disease progression and events not directly related to treatment and/or HK incidence (i.e., MACE, hospitalization, changes in RAASi use and mortality). Data used to inform modelled disease progression and event incidence were primarily sourced from the published literature.

Baseline event rates or probabilities were sourced from published literature for CKD/HF comorbidities where possible; this baseline rate/probability may be modified according RAASi use. Where necessary, the estimated probability of each event is converted to the appropriate cycle length (see Section **Error! Reference source not found.**).

Table 12: Summary of methods employed to model disease progression and events

Population	Events	Baseline incidence rate	Modified by RAASi use?		Modified by K+ levels?	
**CKD**	Progression	Monthly transition probabilities	✓	Any versus none	✗	
	HK	Incidence of K+ above threshold	✓	Any versus none	✓	By definition
	RAASi discontinuation	By RAASi dose level	✓✓	By definition	✓	By K+ category
	RAASi down-titration	By RAASi dose level	✓✓	By definition	✓	By K+ category
	MACE	By CKD stage **	✓	IRR any versus none	✓	By K+ category
	Hospitalization	By CKD stage	✗	Appropriate data not identified	✓	By K+ category
	Mortality*	By CKD stage	✓	IRR any versus none	✓	By K+ category
**HF**	Progression (NYHA)	Monthly transition probabilities	✗	Appropriate data not identified	✗	
	HK	Incidence of K+ above threshold	✓	Any versus none	✓	By definition
	RAASi discontinuation	By RAASi dose level	✓✓	By definition	✓	By K+ category
	RAASi down-titration	By RAASi dose level	✓✓	By definition	✓	By K+ category
	MACE	By RAASi dose level	✓✓	Rate for each dose	✓	By K+ category
	Hospitalization	By serum K+ category; CPRD	✓✓	OR for each dose versus none	✓	By K+ category
	Mortality*	By NYHA class and other risk factors, Seattle Heart Failure Model (SHFM).[2]	✓	Any versus none; Risk factor in SHFM	✓	By K+ category

CKD: chronic kidney disease; CPRD: Clinical Practice Research Datalink; eGFR: estimated glomerular filtration rate; HK: hyperkalemia; HR: hazard ratio; IRR: incidence rate ratio, MACE: major adverse cardiac event; OR: odds ratio; SHFM: Seattle Heart Failure Model

RAASi functionality: ✓✓=modified by RAASi use and dose level, ✓=modified by RAASi use (any), ✗ no functionality
K+ functionality: ✓=functionality ✗ no functionality
Grey shading indicates events/relationships not modelled due to functionality (✗) or due to paucity of identified data
*The higher probability based on (A) comorbidity, RAASi use and K+ levels or (B) life tables is applied throughout
**Cardiovascular event defined as hospitalization for coronary heart disease, heart failure, ischemic stroke, and peripheral arterial disease

6.1. CKD-specific risk/probability inputs

Base case model inputs, relating to the incidence of events that are specific to CKD patients, are presented in **Table 13–Table 15**. It is assumed that the baseline event rates presented in **Table 13** represent risk associated with the reference categories utilized in studies reporting incident rate ratios (IRRs) and odds ratios (ORs) (e.g., normokalaemia or no RAASi use).

Table 13: Baseline MACE, hospitalization and mortality event rate in CKD patients, by CKD stage

**Annual event rate mean (SE*)**	**CKD subgroup**			**Source**
	**3**	**4**	**5**	
MACE^	0.0505 (0.0050)	0.2180 (0.0218)	0.3660 (0.0366)	Go et al.[10]
Hospitalization*	0.0731 (0.0138)	0.1562 (0.0252)	0.2530 (0.0489)	Cozzolino et al.[4]
Mortality (all cause)**	0.0315 (0.0093)	0.0522 (0.0155)	0.1414 (0.0313)***	

CKD: chronic kidney disease; MACE: major adverse cardiac event.
^CKD stage 3 event rates derived from weighted average of CKD stage 3a and 3b, and SE assumed as 10% of mean
* Total number of events observed over 36 months converted to annual rate after simple adjustment for mortality events
** Total number of events observed over 36 months converted to annual rate
Cardiovascular events defined in Go et al.[10] as hospitalization for coronary heart disease, heart failure, ischemic stroke, and peripheral arterial disease
*** Data from Go et al.[10] used in place of Cozzolino et al.[4] as CKD 5 death rate implausibly low.

Table 14: Influence of RAASi on MACE, hospitalization and mortality event rate in CKD patients

**Parameter**	**Mean**	**SE**	**Source**
**Mortality**			
IRR RAASi vs no RAASi	0.179†	0.069*	Linde et al.[9]
**MACE**			
IRR RAASi vs no RAASi	0.621†	0.054*	Linde et al.[9]
**Hospitalization**			
IRR RAASi vs no RAASi	1	0	Null value; no evidence identified

CKD: chronic kidney disease; IRR: incidence rate ratio; MACE: major adverse cardiac event; RAASi: renin-angiotensin-aldosterone system inhibitor; SE: standard error

*SE estimated from 95% confidence interval
†Assumes that RAASi use >50% dose versus <50% dose is reflective of RAASi versus no RAASi

Table 15: Influence of HK on MACE, hospitalization and mortality event rate in CKD patients

Parameter	Mean	SE*	Source
**Mortality**			
IRR Normokalaemia: K+ ≤5	1.000	0.000	Luo et al.[11]
IRR K+ >5 to ≤5.5	1.140	0.056	
IRR K+ >5.5 to ≤6	1.600	0.130	
IRR K+ >6	3.310	0.464	
**MACE****			
IRR Normokalaemia: K+ ≤5	1.000	0.000	Luo et al.[11]
IRR K+ >5 to ≤5.5	1.010	0.020	
IRR K+ >5.5 to ≤6	1.120	0.038	
IRR K+ >6	1.880	0.117	
**Hospitalization**			
IRR CKD stage 3 - Normokalaemia: K+ ≤5	1.000	0.000	Luo et al.[11] (CKD stage 3 conservatively assumed as eGFR 40-49mL/min/1.73m^2 in study)
IRR CKD stage 3 - K+ >5 to ≤5.5	1.070	0.089	
IRR CKD stage 3 - K+ >5.5 to ≤6	1.230	0.179	
IRR CKD stage 3 - K+ >6	1.910	0.564	
IRR CKD stage 4/5 - Normokalaemia: K+ ≤5	1.000	0.000	
IRR CKD stage 4/5 - K+ >5 to ≤5.5	1.000	0.102	
IRR CKD stage 4/5 - K+ >5.5 to ≤6	1.340	0.179	
IRR CKD stage 4/5 - K+ >6	3.650	0.584	

CKD: chronic kidney disease; IRR: incidence rate ratio; MACE: major adverse cardiac event; RAASi: renin-angiotensin-aldosterone system inhibitor; SE: standard error
*SE estimated from 95% confidence interval

6.2. HF-specific risk/probability inputs

Base case model inputs, relating to the incidence of events that are specific to HF patients, are presented in **Table 16-Table 18**. It is assumed that the baseline event rates presented in **Table 16** represent risk associated with the reference categories utilized in studies reporting incident rate ratios (IRRs) and odds ratios (ORs) (e.g., normokalaemia or no RAASi use).

Table 16: Baseline MACE event rates and hospitalization probabilities in HF patients

Parameter	Mean	SE*	Source
**Annual MACE event rate**			
Discontinued RAASi	0.2904	0.0040*	Assumed as RAASi sub-max
RAASi max	0.1485	0.0030*	Linde et al.[9]
RAASi sub-max	0.2904	0.0040*	
**Monthly probability of hospitalization**			
NYHA I	0.0152	0.0015**	Ford et al.[12]
NYHA II	0.0240	0.0024**	
NYHA III	0.0240	0.0024**	
NYHA IV	0.1540	0.0154**	

MACE: major adverse cardiac event; NYHA; New York Heart Association stage; RAASi: renin-angiotensin-aldosterone system inhibitor; SE: standard error
*SE estimated from 95% confidence intervals
**SE assumed as 10% of mean

Table 17: Influence of RAASi on hospitalization in HF patients

Parameter	Mean	SE	Source
**Hospitalization**			
OR RAASi vs no RAASi	0.670	0.033	Flather et al.[13]
OR Sub RAASi vs no RAASi	0.835	0.033	Assumption†

HF: heart failure; OR: odds ratio; RAASi: renin-angiotensin-aldosterone system inhibitor; SE: standard error
†Mean assumed to be 50% impact of maximum dose RAASi, with equal SE

Table 18: Influence of HK on MACE, hospitalization and mortality event rate in CKD patients

Parameter	Mean	SE*	Source
**Mortality**			
HR Normokalaemia: K+ ≤5	1.000	0.000	Krogager et al.[14]
HR K+ >5 to ≤5.5	1.290	0.318	
HR K+ >5.5 to ≤6	3.613	0.973	
HR K+ >6	3.613	0.973	
**MACE****			
IRR Normokalaemia: K+ ≤5	1.000	0.000	Assumed as for CKD patients (Luo et al.[11])
IRR K+ >5 to ≤5.5	1.010	0.020	
IRR K+ >5.5 to ≤6	1.120	0.038	
IRR K+ >6	1.880	0.117	
**Hospitalization**			
IRR CKD stage 3 - Normokalaemia: K+ ≤5	1.000	0.000	Assumed as for CKD patients (Luo et al.[11])
IRR CKD stage 3 - K+ >5 to ≤5.5	1.070	0.089	
IRR CKD stage 3 - K+ >5.5 to ≤6	1.230	0.179	
IRR CKD stage 3 - K+ >6	1.910	0.564	
IRR CKD stage 4/5 - Normokalaemia: K+ ≤5	1.000	0.000	
IRR CKD stage 4/5 - K+ >5 to ≤5.5	1.000	0.102	
IRR CKD stage 4/5 - K+ >5.5 to ≤6	1.340	0.179	
IRR CKD stage 4/5 - K+ >6	3.650	0.584	

CKD: chronic kidney disease; HR: hazard ratio; IRR: incidence rate ratio; MACE: major adverse cardiac event; OR: odds ratio; RAASi: renin-angiotensin-aldosterone system inhibitor; SE: standard error
*SE estimated from 95% confidence interval

Seattle Heart Failure Model

Mortality in the HF population is modelled via implementation of the SHFM[2]: a multivariate Cox model for survival among HF patients. Coefficient estimates of the SHFM are presented in **Table 19**. Since the use of ACE inhibitors and ARBs are among the predictive factors of the SHFM, the proportion of RAASi users that are on ACE and ARB therapy are included in the baseline characteristics.

Table 19: SHFM for survival in HF patients[2]

Explanatory variable	Hazard ratio		
	**Mean**	**95% CI**	**SE***
Age (years/10)	1.09	(0.985–1.205)	0.0561
Male sex	1.089	(0.839–1.414)	0.1467
NYHA (1–4)	1.6	(1.019–2.511)	0.3806
100/Ejection fraction	1.03	(1.010–1.050)	0.0102
Ischemic etiology (0/1)	1.354	(1.074–1.707)	0.1615
SBP (mmHg/10)	0.877	(0.823–0.935)	0.0286
Diuretic dose (mg/kg)	1.178	(1.097–1.266)	0.0431
Allopurinol use (0/1)	1.571	(1.170–2.109)	0.2395
Statin use (0/1)	0.63	(0.410–0.978)	0.1449
If sodium<138, 138-sodium	1.05	(1.005–1.097)	0.0235
Cholesterol (100/mg/dL)	2.206	(1.045–4.656)	0.9212
If hemoglobin <16, 16-hemoglobin	1.124	(1.053–1.200)	0.0375
If hemoglobin >16, hemoglobin-16	1.336	(1.010–1.767)	0.1931
Lymphocytes (%/5)	0.897	(0.846–0.951)	0.0523
Uric acid (mg/dL)	1.064	(1.022–1.108)	0.0219
ACE use (0/1)	0.77	-	0.0770
Beta blocker use (0/1)	0.66	-	0.0660
ARB use (0/1)	0.85	-	0.0850
K-sparing diuretic use (0/1)	0.74	-	0.0740
ICD (0/1)	0.73	-	0.0730
BICD (0/1)	0.79	-	0.0790

ACE: angiotensin converting enzyme; ARB: angiotensin receptor blocker; BICD: Biventricular implantable cardioverter-defibrillator; NYHA: New York Heart Association classification; ICD: Implantable cardioverter-defibrillator
*SE estimated from 95% confidence interval, or assumed as 10% of mean where CI not presented

6.3. Life tables

Life tables are utilized to describe all-cause mortality experienced in the general population, where each row contains the probability that an individual aged x will die by age x+1. National life tables are typically available in this form, or similar, up to age 100. After this age, individuals are assumed to survive no longer; in other words, modelled individuals that survive to age 101 die in the next modelled cycle (**Table 20**).

Table 20: Excerpt from 2021 Italian life tables

Age	Annual probability of death	
	Male	Female
65	0.01174	0.00616
66	0.01294	0.00677
67	0.01429	0.00754
...	...	…
98	0.33660	0.29474
99	0.35983	0.32189
100	0.39031	0.35223
101	*1.00000*	*1.00000*
Source: Italian National Institute of Statistics [15]		

7. Treatment duration, discontinuation and retreatment

Modelled patients receive treatment (Patiromer) from the beginning of the simulation until one of the following occurs:

A. Patient reaches ESRD and commences RRT
B. Modelled K+ level falls below a user-defined acceptable range for discontinuation of treatment
C. Discontinuation due to other reasons, defined by a monthly probability of discontinuation

Following discontinuation due to any reason, patients incur HK and RAASi discontinuation/down-titration risk in line with the SoC arm. SoC may not be discontinued.

The model also simulates repeat treatment in patients that discontinue Patiromer due to reasons B or C above. Repeat treatment is initiated when the patient’s modelled K+ level rises above 5.5 mmol/L. If treatment is repeated, K+ is modelled according to the patiromer profile specified for the first month of treatment.

Figure 1 presents the logical process followed to simulate treatment discontinuation (patiromer) and repeat treatment.

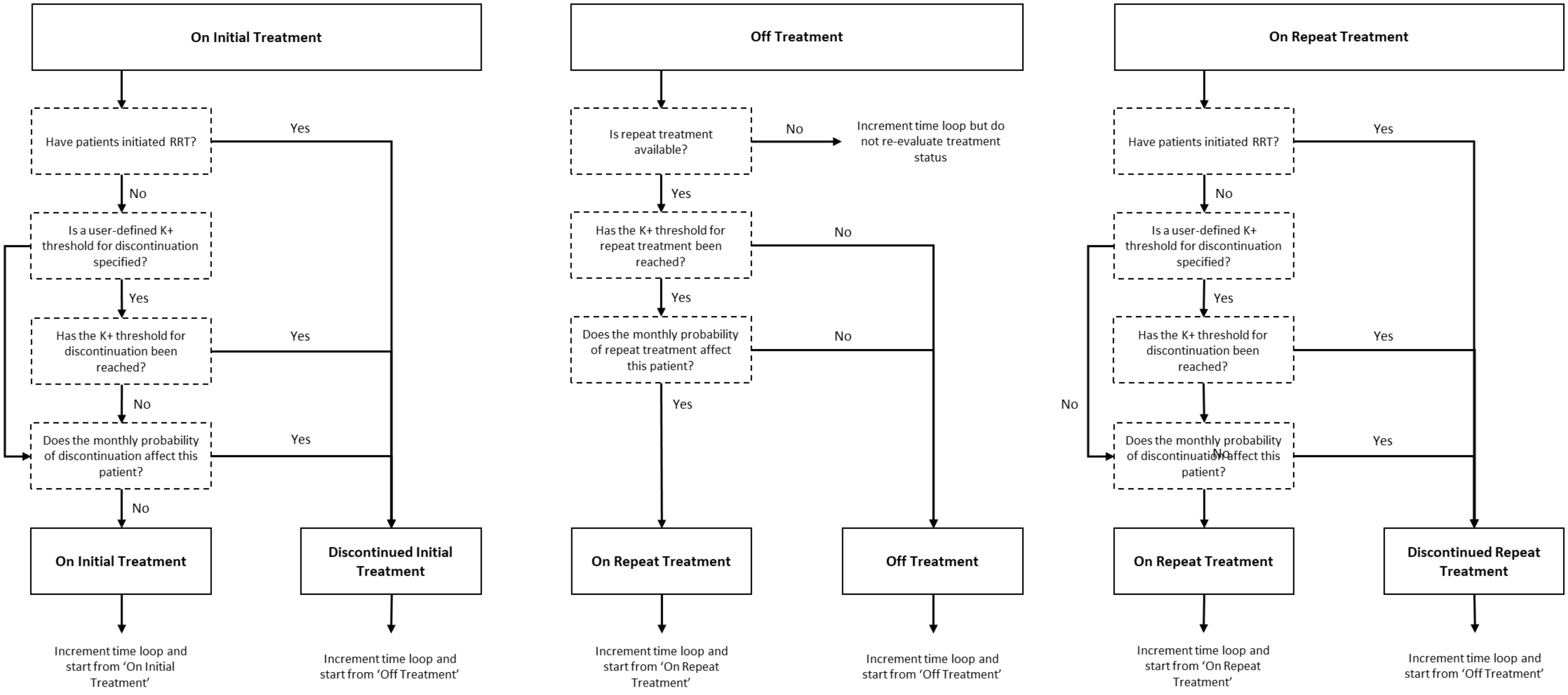

Figure 1: Treatment discontinuation and re-initiation algorithm (individual patient perspective)

7.1. K+ threshold for repeat treatment

Discontinued patients may initiate repeat treatment after the first cycle if their modelled K+ value rises above 5.5 mmol/L (prior to the initiation of RRT). Repeat treatment corresponds to the initial patiromer treatment; however, in a simplified form, only the input values relating to month 1 of initial treatment are used (i.e., treatment response after 1 month, RAASi status after 1 month and HK event rates during the first month of treatment). The K+ threshold for repeat treatment is applied inclusive of the threshold value, such that repeat treatment is modelled if K+ equals or exceeds the threshold value. Repeat treatment may occur multiple times.

7.2. Monthly probability of discontinuation

Modelled patients are subject to a monthly probability of discontinuing treatment. Monthly discontinuation probabilities for patiromer were estimated using available data, as summarized in **Table 21**. Since SoC comprises a combination of lifestyle interventions and routine HK management, the probability of discontinuation from SoC is assumed to be 0. For Patiromer, the annual probability of discontinuation was estimated using data from the extended phase of the OPAL-HK trial[1].

Table 21: Monthly probability of discontinuation

	Mean (%)	SE (%)	Source
Patiromer	10.33*	4.10	OPAL-HK CSR[1]
* Extended phase probability, with 10/55 patients discontinuing during the 8-week period, adjusted to monthly probability			

8. Notes on the application of odds ratios and hazard ratios

Odds ratios (ORs)

Where ORs are applied in the model, for consistency they are applied to annual probabilities. Probabilities are converted to odds using the formula: odds = probability / (1 – probability). The odds ratio is subsequently multiplied by the odds, and the resultant odds is converted back to a probability using the formula: probability = odds / (1 + odds).

The resultant probability may then be converted to the necessary time frame (e.g., monthly cycle).

Hazard ratios (HRs)

Hazard ratios are applied directly to rates. If inputs are loaded as probabilities, they are converted to rates before applying the hazard ratio and are then converted back to probabilities.

Probability / rate conversion

Probabilities are converted to rates using the following formula: p = 1 – Exp(- r * t)

Rates are converted to probabilities using the following formula: r = -ln(1 – p) / t

In both the above, p = probability; r = rate; t = time.

References

1. Vifor Pharma OPAL-HK CSR. Data on file. (2014).
2. Levy WC, Mozaffarian D, Linker DT, Sutradhar SC, Anker SD, Cropp AB, Anand I, et al. (2006) The Seattle Heart Failure Model prediction of survival in heart failure. Circulation;113(11):1424-33.
3. Xie X, Liu Y, Perkovic V, Li X, Ninomiya T, Hou W, Zhao N, et al. (2016) Renin-angiotensin system inhibitors and kidney and cardiovascular outcomes in patients with CKD: a Bayesian network meta-analysis of randomized clinical trials. American Journal of Kidney Diseases;67(5):728-41.
4. Cozzolino M, Bolasco P, Ronco C, Conte G, Menè P, Mereu MC, Di Luca M, et al. (2018) Clinical Management of Chronic Kidney Disease Patients in Italy: Results from the IRIDE Study. Nephron;140(1):39-47. doi:10.1159/000490769.
5. Italian Society of Nephrology Italian Dialysis and Transplant Registry Report 2019(2019) December 2022. Available from: https://ridt.sinitaly.org/2021/10/12/report-2019/.
6. Italian National Transplant Centre (Centro Nazionale Trapianti) Assessment of quality of the activity of kidney transplant 2000-2019(2019) December 2022. Available from: https://trapianti.sanita.it/statistiche/attivita/2021_D_QUALITA_ORGANI_RENE_00-19.pdf.
7. UK Renal Association. UK Renal Registry 23rd Annual Report 2021. (2021) February 2021. Available from: https://renal.org/about-us/who-we-are/uk-renal-registry.
8. Yao G, Freemantle N, Calvert MJ, Bryan S, Daubert J-C, Cleland JG (2007) The long-term cost-effectiveness of cardiac resynchronization therapy with or without an implantable cardioverter-defibrillator. European heart journal;28(1):42-51.
9. Linde C, Bakhai A, Furuland H, Evans M, McEwan P, Ayoubkhani D, Qin L (2019) Real-World Associations of Renin-Angiotensin-Aldosterone System Inhibitor Dose, Hyperkalemia, and Adverse Clinical Outcomes in a Cohort of Patients With New-Onset Chronic Kidney Disease or Heart Failure in the United Kingdom. J Am Heart Assoc;8(22):e012655. doi:10.1161/jaha.119.012655.
10. Go AS, Chertow GM, Fan D, McCulloch CE, Hsu C-y (2004) Chronic kidney disease and the risks of death, cardiovascular events, and hospitalization. New England Journal of Medicine;351(13):1296-305.
11. Luo J, Brunelli SM, Jensen DE, Yang A (2016) Association between serum potassium and outcomes in patients with reduced kidney function. Clin J Am Soc Nephrol;11(1):90-100.
12. Ford E, Adams J, Graves N (2012) Development of an economic model to assess the cost-effectiveness of hawthorn extract as an adjunct treatment for heart failure in Australia. BMJ Open;2(5):e001094. doi:10.1136/bmjopen-2012-001094.
13. Flather MD, Yusuf S, Køber L, Pfeffer M, Hall A, Murray G, Torp-Pedersen C, et al. (2000) Long-term ACE-inhibitor therapy in patients with heart failure or left-ventricular dysfunction: a systematic overview of data from individual patients. ACE-Inhibitor Myocardial Infarction Collaborative Group. Lancet;355(9215):1575-81. doi:10.1016/s0140-6736(00)02212-1.
14. Krogager ML, Eggers-Kaas L, Aasbjerg K, Mortensen RN, Køber L, Gislason G, Torp-Pedersen C, et al. (2015) Short-term mortality risk of serum potassium levels in acute heart failure following myocardial infarction. European Heart Journal-Cardiovascular Pharmacotherapy:pvv026.
15. Italian National Institute of Statistics Population and Households: Life Tables 2021(2022) December 2022. Available from: http://dati.istat.it/Index.aspx?QueryId=19053&lang=en.
