# Supplementary material for: Hyperkalemia in chronic kidney disease patients with and without heart failure: an Italian economic modelling study
Source: Cost Eff Resour Alloc. 2024 May 21;22:42. doi: 10.1186/s12962-024-00547-y (PMC11106859; doi:10.1186/s12962-024-00547-y)
Supplement: Supplementary file 2 — Additional file 2: Disease progression and events. Provides details of the disease progression and events utilized in the model [file 12962_2024_547_MOESM2_ESM.pdf]

Additional file 2

This appendix provides details of disease progression and events utilized in the model.

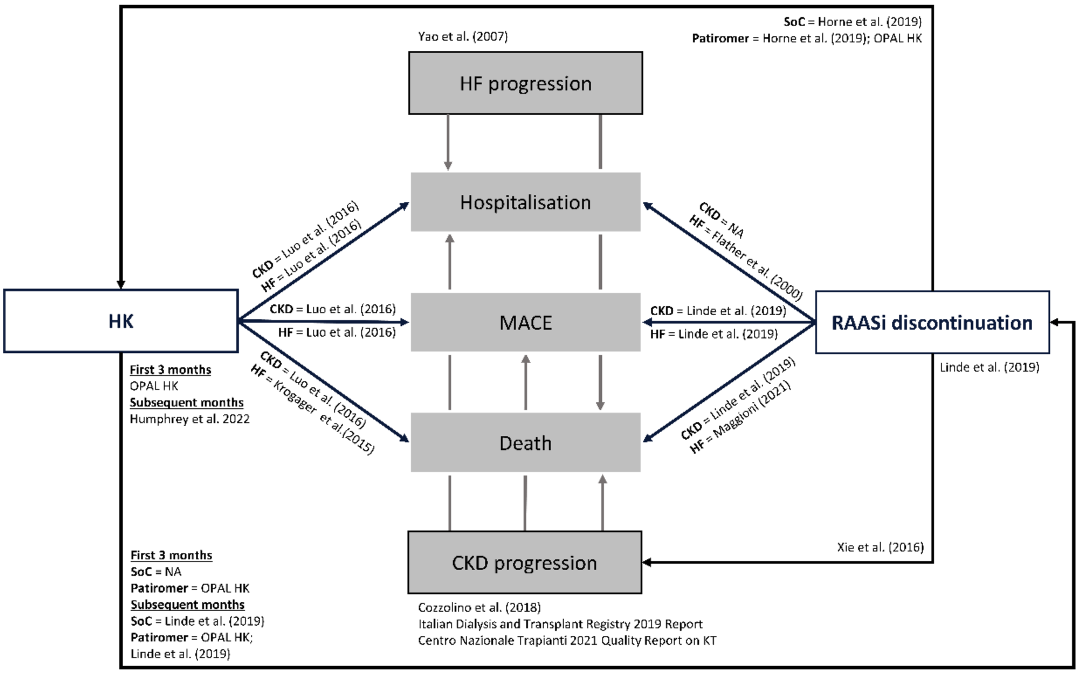

Fig. 1 Influence of RAASi use on disease progression and events

Notes: References below each box describe the baseline probabilities/rates; references alongside arrows describe the influence of one disease component on the other, with influences applied to the baseline probabilities/rates. **Abbreviations:** CKD: chronic kidney disease; HF: heart failure; HK: hyperkalemia; KT: kidney transplant; MACE: major adverse cardiac event; NA: not applicable; NHYA: New York Heart Association; RAASi: renin–angiotensin–aldosterone system inhibitor; SoC: standard of care.
