# Supplementary material for: Hyperkalemia in chronic kidney disease patients with and without heart failure: an Italian economic modelling study
Source: Cost Eff Resour Alloc. 2024 May 21;22:42. doi: 10.1186/s12962-024-00547-y (PMC11106859; doi:10.1186/s12962-024-00547-y)
Supplement: Supplementary file 3 — Additional file 3: Costs. Provides detail of cost data stylized in the model. All costs are presented in 2021 EUR [file 12962_2024_547_MOESM3_ESM.pdf]

Additional file 3

This appendix provides details of cost data utilized in the model. All costs are presented in 2021 EUR.

1. Health state and event costs

Table 1 summarizes the direct costs applied to modelled health states and events. Where more than one event can occur within a patient's lifetime (e.g., hospitalization events or MACE), the same costs are applied to initial and subsequent events.

Table 1: Health state and event costs

**Parameter**	**Mean (€)**	**SE**	**Source**
Annual cost CKD 3	2,227.98	146.22	Jommi et al.[1]; CKD stage 3a and CKD stage 3b estimates combined through weighted average; cost inflated from 2014 values†
Annual cost CKD 4	4,353.68	406.29	Jommi et al.[1]; cost inflated from 2014 values†
Annual cost CKD 5 (pre-RRT)	5,724.77	625.18	Jommi et al.[1]; cost inflated from 2014 values†
Annual cost of dialysis	33,532.46	476.89	Roggeri et al.[2]; weighted by dialysis type observed in publication; hospitalization cost subtracted as assumed to be captured elsewhere; cost inflated from 2014 values†
Dialysis access cost	5,779.33	577.93*	Italian Ministry of Health[3]; Nomenclature code 38.95 - Venous catheterization for renal dialysis (inflated from 2018 costs) and DRG 120 - Other circulatory system operative procedures (inflated from 2019 costs). Weighted by dialysis modality in Roggeri et al.[4]
One-off cost of dialysis complications	0	0	Assumed to be captured in annual dialysis costs
One-off transplant procedure cost	22,197.94	2,219.79*	Roggeri et al.[4]; cost inflated from 2017 values†
Annual cost of transplant maintenance	19,077.11	1,797.73	Roggeri et al.[4]; hospitalization and surgery cost subtracted as assumed to be captured elsewhere; cost inflated from 2017 values†
NYHA I	0	0	Assumed to be captured within CKD health state costs and event costs
NYHA II	0	0	
NYHA III	0	0	
NYHA IV	0	0	
Event cost: MACE	4,464.59	446.46*	Corrao et al.[5]; cost derived from index MACE event; cost inflated from 2011 values†
Event cost: Hospitalization	3,617.77	361.78*	Roggeri et al.[6]; cost inflated from 2012 values†
Event cost: RAASi discontinuation	84.40	8.44*	**Table 4**
Event cost: RAASi down-titration	126.60	12.66*	**Table 2**
Event cost: return to maximum RAASi use	36.92	3.69*	**Table 3**

CKD: chronic kidney disease; MACE, Major adverse cardiac event; NHYA: New York Heart Association; RRT: renal replacement therapy.
*SE values assumed 10% of the mean.
†See Table 10 for inflation indices.

RAASi discontinuation costs

The derivation of RAASi discontinuation costs is detailed in **Table 2** to **Table 4.**

Table 2. RAASi dose down-titration

Resource	Proportion use (%)	Resource use	Unit Cost	Source	Total cost
**Secondary care costs**					
Outpatient visit	90.00	3.00	€26.92	Riccio et al.[7]; Hourly cost for a nephrologist, assuming a 30 minute visit; cost inflated from 2018 values†	€72.67
Inpatient day	10.00	3.00	€79.77	Italian Ministry of Health[3]; DRG code 145 - Other diagnosis of the circulatory system without complications - admissions of day (inflated from 2019 costs).	€23.93
U&E test	100.00	3.00	€10.00	Assumption	€30.00
**Total weighted secondary care costs**					**€126.60**
U&E: urea and electrolytes					

Table 3. RAASi dose up-titration cost

Resource	Proportion use (%)	Resource use	Unit Cost	Source	Total cost
**Secondary care costs**					
Outpatient visit	100.00	1.00	€26.92	Riccio et al.[7]; Hourly cost for a nephrologist, assuming a 30 minute visit; cost inflated from 2018 values†	€26.92
U&E test	100.00	1.00	€10.00	Assumption	€10.00
**Total weighted secondary care costs**					**€36.92**
U&E: urea and electrolytes					

Table 4. RAASi discontinuation cost

Resource	Proportion use (%)	Resource use	Unit Cost	Source	Total cost
**Secondary care costs**					
Outpatient visit	90.00	2.00	€26.92	Riccio et al.[7]; Hourly cost for a nephrologist, assuming a 30 minute visit; cost inflated from 2018 values†	€48.45
Inpatient day	10.00	2.00	€79.77	Italian Ministry of Health[3]; DRG code 145 - Other diagnosis of the circulatory system without complications - admissions of day (inflated from 2019 costs).	€15.95
U&E test	100.00	2.00	€10.00	Assumption	€20.00
**Total weighted secondary care costs**					**€84.40**
U&E: urea and electrolytes					

2. RAASi therapy costs

Costs associated with ongoing RAASi therapy (ACE, ARB and/or mineralocorticoid receptor agonist [MRA]) are applied at two levels, corresponding to “max” and “sub-max” levels referenced elsewhere. Annual costs for the two categories are derived from a series of user-defined inputs describing the proportions of patients receiving each therapy and average doses under “max” and “sub-max” therapy. These are combined with unit costs per mg, to derive weighted average costs per patient receiving RAASi therapy at each level.

Table 5 summarizes annual aggregated RAASi costs, whilst **Table 6** and **Table 7** describe their derivation in more detail.

Table 5: Costs applied to RAASi use

Parameter	Mean (€)	SE	Source
Annual cost of RAASi: Optimal therapy (Max)	115.58	11.56	See Table 6 and Table 7
Annual cost of RAASi: Sub-optimal therapy (Sub-max)	57.79	5.78	

Table 6: Weighted annual RAASi cost – Optimal therapy

Agent	Percentage of cohort	Source	Average daily dose (mg)	Source	Cost per mg	Mediately[8] drug name
**ACE inhibitor**						
Ramipril	67.5%	OPAL-HK[9]; ramipril assumed representative	10.00	ESC[10]	€0.02482	RAMIPRIL (ARISTO) 10 mg compress
**ARB**						
Candesartan	39.1%	OPAL-HK[9]; candesartan assumed representative	32.00	ESC[10]	€0.01037	'CANDESARTAN (DOC GENERICI) 32 mg compress
**MRA**						
Spironolactone BP	7.3%	OPAL-HK[9]; spironolactone assumed representative	50.00	ESC[10]	€0.00525	ALDACTONE 25 mg capsule rigid
**Weighted average (daily) daily cost**	**€0.31643**					
**Weighted average annual cost**	**€115.58**					

ACE: angiotensin converting enzyme; ARB: Angiotensin II Receptor Blockers; MRA: Mineralocorticoid Receptor Antagonist; RAASi: renin–angiotensin–aldosterone system inhibitor.
Weighted average daily cost: Average daily dose (mg) x Cost per mg x Percentage of cohort on Drug; Weighted average annual cost: Weighted average daily cost x 365.25

Table 7: Weighted annual RAASi cost – Sub-optimal dosing

Agent	Percentage of cohort	Source	Average daily dose (mg)*	Source	Cost per mg	Mediately[8] drug name
**ACE inhibitor**						
Ramipril	67.5%	OPAL-HK[9]; ramipril assumed representative	5.00	ESC[10]	€0.02482	RAMIPRIL (ARISTO) 10 mg compress
**ARB**						
Candesartan	39.1%	OPAL-HK[9]; candesartan assumed representative	16.00	ESC[10]	€0.01037	'CANDESARTAN (DOC GENERICI) 32 mg compress
**MRA**						
Spironolactone BP	7.3%	OPAL-HK[9]; spironolactone assumed representative	25.00	ESC[10]	€0.00525	ALDACTONE 25 mg capsule rigid
**Weighted average (daily) daily cost**	**€0.15821**					
**Weighted average annual cost**	**€57.79**					

ACE: angiotensin converting enzyme; ARB: Angiotensin II Receptor Blockers; MRA: Mineralocorticoid Receptor Antagonist; RAASi: renin–angiotensin–aldosterone system inhibitor.
*Assumed as half daily recommended dose
Weighted average daily cost: Average daily dose (mg) x Cost per mg x Percentage of cohort on Drug; Weighted average annual cost: Weighted average daily cost x 365.25

3. HK treatment costs

Costs associated with the management of HK events in the maintenance phase have been defined for two K+ thresholds: K+ >5.5 to ≤6 mmol/L and K+ >6.0 mmol/L. These costs are based on expected resource use for HK at each level and are applied during the month of incidence only. The cost inputs are summarized in **Table 8** (see **Table 9** for a more detailed derivation).

Table 8. HK event costs

Resource	Mean	SE	Source
Moderate HK event: K+ >5.5 to ≤6 mmol/L	73.83	7.38*	**Table 9**
Severe HK event: K+ >6.0 mmol/L	1,916.11	191.61*	**Table 9**
HK: hyperkalemia; SE: standard error<br>*SE assumed as 10% of mean			

Table 9. HK event cost

Resource	Percentage (%)	Unit	Unit Cost	Source	Total cost
**Moderate HK: K+ >5.5 to ≤6 mmol/L**					
Outpatient visit	100.00	2.00	€26.92	Riccio et al.[7]; Hourly cost for a nephrologist, assuming a 30 minute visit; cost inflated from 2018 values†	€53.83
U&E test	100.00	2.00	€10.00	Assumption	€20.00
**Total moderate HK cost**					**€73.83**
**Severe HK: K+ >6.0 mmol/L**					
Inpatient day	100.00	5.40	€351.84	Italian Ministry of Health[3]; DRG code 145 - Other diagnosis of the circulatory system without complications - admissions of day (inflated from 2019 costs).	€1,899.92
ECG	100.00	1.00	€12.35	Assumption	12.35
Insulin	100.00	1.00	€1.18	Mediately[8]; HUMULIN I 100 ml/ml sospensione iniettabile 1000UI 10 ml; assumed as proxy at 1 unit/kg per day, with average weight of 80kg	€1.18
Glucose	100.00	1.00	€0.45	Mediately[8]; GLUCOSIO (BAXTER) 50 % soluzione iniettabile	€0.45
Calcium gluconate	100.00	2.00	€1.11	Mediately[8]; CALCIO GLUCONATO (B.BRAUN MILANO) 10 % soluzione iniettabile	€2.22
**Total severe HK cost**					**€1,916.11**
ECG: electrocardiogram; HK: hyperkalemia; U&E: urea and electrolytes.					

4. Patiromer and SoC costs

The cost of Patiromer treatment is based on an 8.4g daily dose costing €10.81 per day. SoC costs are assumed to be €0.00 and accounted for in the HK event costs.

5. Inflation factors and inflated cost calculations

Inflation factors were calculated using The World Bank consumer price indices (Table 10)[11]. Inflation factors were used to inflate costs to 2021 values.

Table 10: Inflation factors

Year	Annual CPI inflation	Inflation factors	Compared to 2021
1990	6.4566	1.0646	2.1456
1991	6.2500	1.0625	2.0155
1992	5.2706	1.0527	1.8969
1993	4.6267	1.0463	1.8019
1994	4.0518	1.0405	1.7222
1995	5.2354	1.0524	1.6552
1996	4.0070	1.0401	1.5728
1997	2.0431	1.0204	1.5122
1998	1.9551	1.0196	1.4820
1999	1.6635	1.0166	1.4535
2000	2.5377	1.0254	1.4298
2001	2.7852	1.0279	1.3944
2002	2.4653	1.0247	1.3566
2003	2.6726	1.0267	1.3240
2004	2.2067	1.0221	1.2895
2005	1.9853	1.0199	1.2617
2006	2.0908	1.0209	1.2371
2007	1.8297	1.0183	1.2118
2008	3.3478	1.0335	1.1900
2009	0.7748	1.0077	1.1514
2010	1.5255	1.0153	1.1426
2011	2.7806	1.0278	1.1254
2012	3.0414	1.0304	1.0950
2013	1.2200	1.0122	1.0626
2014	0.2410	1.0024	1.0498
2015	0.0388	1.0004	1.0473
2016	-0.0940	0.9991	1.0469
2017	1.2265	1.0123	1.0479
2018	1.1375	1.0114	1.0352
2019	0.6112	1.0061	1.0236
2020	-0.1377	0.9986	1.0173
2021	1.8738	1.0187	1.0187

CPI: consumer price index

References

1. Jommi C, Armeni P, Battista M, di Procolo P, Conte G, Ronco C, Cozzolino M, et al. (2018) The Cost of Patients with Chronic Kidney Failure Before Dialysis: Results from the IRIDE Observational Study. Pharmacoecon Open;2(4):459-67. doi:10.1007/s41669-017-0062-z.
2. Roggeri A, Roggeri DP, Zocchetti C, Bersani M, Conte F (2017) Healthcare costs of the progression of chronic kidney disease and different dialysis techniques estimated through administrative database analysis. J Nephrol;30(2):263-9. doi:10.1007/s40620-016-0291-8.
3. Italian Ministry of Health Annual report on hospitalization activity (SDO data 2019)(2021) December 2022. Available from: https://www.salute.gov.it/portale/documentazione/p6_2_2_1.jsp?id=3002&lingua=italiano.
4. Roggeri DP, Roggeri A, Zocchetti C, Cozzolino M, Rossi C, Conte F (2019) Real-world data on healthcare resource consumption and costs before and after kidney transplantation. Clin Transplant;33(10):e13728. doi:10.1111/ctr.13728.
5. Corrao G, Ghirardi A, Ibrahim B, Merlino L, Maggioni AP (2014) Burden of new hospitalization for heart failure: a population-based investigation from Italy. Eur J Heart Fail;16(7):729-36. doi:10.1002/ejhf.105.
6. Roggeri DP, Roggeri A, Salomone M (2014) Chronic Kidney Disease: Evolution of Healthcare Costs and Resource Consumption from Predialysis to Dialysis in Piedmont Region, Italy. Advances in Nephrology;2014:680737. doi:10.1155/2014/680737.
7. Riccio E, Sabbatini M, Capuano I, Pellegrino AM, Petruzzelli LA, Pisani A (2020) Oral Sucrosomial® iron versus intravenous iron for recovering iron deficiency anaemia in ND-CKD patients: a cost- minimization analysis. BMC Nephrology;21(1):57. doi:10.1186/s12882-020-01716-w.
8. Modra JagodaMediately (2022). Available from: https://mediately.co/it.
9. Vifor Pharma OPAL-HK CSR. Data on file. (2014).
10. Ponikowski P, Voors AA, Anker SD, Bueno H, Cleland JG, Coats AJ, Falk V, et al. (2016) 2016 ESC Guidelines for the diagnosis and treatment of acute and chronic heart failure: The Task Force for the diagnosis and treatment of acute and chronic heart failure of the European Society of Cardiology (ESC) Developed with the special contribution of the Heart Failure Association (HFA) of the ESC. European heart journal;37(27):2129-200.
11. The World BankConsumer price indices (2022). Available from: https://data.worldbank.org/indicator/FP.CPI.TOTL.ZG?end=2021&locations=IT&start=2000.
