# Supplementary material for: Hyperkalemia in chronic kidney disease patients with and without heart failure: an Italian economic modelling study
Source: Cost Eff Resour Alloc. 2024 May 21;22:42. doi: 10.1186/s12962-024-00547-y (PMC11106859; doi:10.1186/s12962-024-00547-y)
Supplement: Supplementary file 4 — Additional file 4: Utility and disutility. Provides details of utility and disutility input parameters utilized in the model [file 12962_2024_547_MOESM4_ESM.pdf]

Additional file 4

This appendix provides details of utility and disutility input parameters utilized in the model.

1. Overview

Health-related utility values are utilized to characterize the quality of life of modelled patients and to estimate quality adjusted life-years (QALYs).

Health state utility values for CKD and NYHA health states are utilized by the model and are applied multiplicatively. For example, a patient in CKD stage 3 and NYHA stage II would have the health state utility of one multiplied by the other.

In contrast, transient events such as HK, hospitalisation and MACE incur disutilities in the cycle of incidence that are applied additively.

2. Health state utility and impact of complications

Health state utility values are applied multiplicatively and scaled to the current cycle length. Transient events are associated with a disutility that is applied additively in the cycle of incidence. Points of note in the application of utility values are as follows:

- If a patient has CKD and HF, both CKD and NYHA utility weights are applied.
- Disutilities associated with HK, hospitalisation and MACE events are applied in the cycle of incidence only (true for all cycles throughout the modelled horizon).
- Following the initiation of RRT, health state utility weights of dialysis and transplant are applied without adjustment for prior HF.

Table 1 summarizes health state utility weights applied according to CKD stage, dialysis, transplant, and NYHA classification and disutility associated with HK, MACE or hospitalisation events and dialysis complications.

Table 1: Health state utilities and disutilities of events

Parameter	Mean	SE	Source
Health state utility: CKD 3	0.870	0.034	Gorodetskaya et al.[1]
Health state utility: CKD 4	0.850	0.029	Gorodetskaya et al.[1]
Health state utility: CKD 5 (pre RRT)	0.570	0.057	Lee et al.[2]
Health state utility: Dialysis	0.451	0.045§	Lee et al.[2]*
Health state utility: Transplant	0.710	0.019	Lee et al.[2]
Health state utility: NYHAI	0.855	0.005	Göhler et al.[3]
Health state utility: NYHAII	0.771	0.005	Göhler et al.[3]
Health state utility: NHYAIII	0.673	0.006	Göhler et al.[3]
Health state utility: NYHAIV	0.532	0.027	Göhler et al.[3]
Disutility: HK event	0.000	0.000	Assumed to be captured in health state utility estimates
Disutility: MACE	-0.050	0.040	Kent et al.[4]
Disutility: Hospitalisation	-0.024	0.007	Göhler et al.[3]
Disutility: Dialysis complications	-0.060	0.006§	NICE CG125[5], Sennfalt et al.[6]

CKD: chronic kidney disease; HF: heart failure; HK: hyperkalemia; MACE, Major adverse cardiac event; NYHA: New York Heart Association; SE: standard error
*Weighted by dialysis modalities reported in the Italian Nephrology Societies 2021 RIDT report (slide 14)[7], utilizing the following calculation: $(0.44 \times 0.55 + 0.53 \times 0.08)/0.63 = 0.451$
§ SE values assumed 10% of mean

References

1. Gorodetskaya I, Zenios S, Mcculloch CE, Bostrom A, Hsu C-Y, Bindman AB, Go AS, et al. (2005) Health-related quality of life and estimates of utility in chronic kidney disease. Kidney international;68(6):2801-8. doi:10.1111/j.1523-1755.2005.00752.x.
2. Lee AJ, Morgan CL, Conway P, Currie CJ (2005) Characterisation and comparison of health-related quality of life for patients with renal failure. Current medical research and opinion;21(11):1777-83. doi:10.1185/030079905X65277. .
3. Göhler A, Geisler BP, Manne JM, Kosiborod M, Zhang Z, Weintraub WS, Spertus JA, et al. (2009) Utility Estimates for Decision–Analytic Modeling in Chronic Heart Failure—Health States Based on New York Heart Association Classes and Number of Rehospitalizations. Value in Health;12(1):185-7. doi:10.1111/j.1524-4733.2008.00425.x.
4. Kent S, Briggs A, Eckermann S, Berry C (2013) Are value of information methods ready for prime time? An application to alternative treatment strategies for NSTEMI patients. International journal of technology assessment in health care;29(04):435-42. doi:10.1017/S0266462313000433. .
5. National Institute for Health and Care Excellence. Clinical guideline [CG125]: Chronic kidney disease (stage 5): peritoneal dialysis(2011) 08 December 2016. Available from: https://www.nice.org.uk/guidance/cg125.
6. Sennfalt K, Magnusson M, Carlsson P (2002) Comparison of hemodialysis and peritoneal dialysis--a cost-utility analysis. Peritoneal Dialysis International;22(1):39-47.
7. Italian Society of Nephrology Italian Dialysis and Transplant Registry Report 2019(2019) December 2022. Available from: https://ridt.sinitaly.org/2021/10/12/report-2019/.
