# Supplementary material for: Hyperkalemia in chronic kidney disease patients with and without heart failure: an Italian economic modelling study
Source: Cost Eff Resour Alloc. 2024 May 21;22:42. doi: 10.1186/s12962-024-00547-y (PMC11106859; doi:10.1186/s12962-024-00547-y)
Supplement: Supplementary file 5 — Additional file 5: Scenario analysis. Provides additional data utilized in scenario analyses [file 12962_2024_547_MOESM5_ESM.pdf]

Additional file 5

This appendix presents the additional data utilized in scenario analyses.

1. Scenario analysis 1

Multi-centric Italian studies have assessed the impact of RAASi use on cardiovascular events and hospitalisation in patients with CKD and HF[1, 2]. In these studies, RAASi use was defined by medication adherence which was calculated as the proportion of days covered (PDC), i.e., the ratio between the number of days of medication and days of observation (365 days) multiplied by 100. Persons with >80% PDC were considered adherent. The hazard rate of clinical events was compared between RAASi nonadherent vs. adherent persons with hyperkalemia (K+ ≥5.5 m/mol). **Table 1** presents the data included in scenario analysis.

Table 1. Hazard ratios for MACE and hospitalisation in RAASi nonadherent vs. adherent patients

Parameter	Hazard ratio	SE*	Source
**Mortality**			
CKD	2.26	0.390	Santoro et al.[1]
HF	2.09	0.167	Volterrani et al.[2]
**MACE**			
CKD	1.45	0.270	Santoro et al.[1]
HF	1.39	0.203	Volterrani et al.[2]
CKD: chronic kidney disease; HF: heart failure; MACE: major adverse cardiac event; RAASi: renin-angiotensin-aldosterone system inhibitor; SE: standard error<br>For RAASi sub-max dose, the used value is conservatively assumed to be consistent with the non-adherent group.			

2. Scenario analysis 2

2.1. Baseline characteristics

In scenario analysis, patient characteristics required to estimate the likelihood of death in patients with HF were informed by Italian sources. These values are specified in **Table 2**.

Table 2. Baseline characteristics available to describe the Italian population

Baseline patient characteristic	Mean	SE	Source
Ejection fraction (mL)	34.85	0.31	Maggioni et al.[3]
Ischemic etiology (%)*	43.48%	1.26%	
Systolic blood pressure (mmHg)	141	0.81	Provenzano et al.[4]
Total cholesterol (mg/dL)	198	2.02	Provenzano et al.[5]
Hemoglobin (g/dL)	12.2	0.07	Provenzano et al.[4]
Percent lymphocytes (%)*	26.00%	1.31%	PRAISE[6]
Sodium (mEq/L)	149.1	2.66	Provenzano et al.[4]
Uric acid (mg/dL)	8.90	0.08	PRAISE[6]
K+ sparing diuretics*	56.3%	4.0%	PRAISE[6]
Beta blockers*	37.2%	2.1%	Provenzano et al.[5]
Statin*	42.9%	2.1%	Provenzano et al.[5]

Baseline patient characteristic	Mean	SE	Source
Allopurinol	10.00%	0.9%	PRAISE[6]
ICD	0.0%	0.0%	
BICD	0.0%	0.0%	
Proportion of RAASi users on ACE inhibitors*	51.8%	2.1%	Provenzano et al.[5]
Proportion of RAASi users on ARB*	46.6%	2.1%	
Diuretic dose (mg/kg)	1.45	0.04	PRAISE[6]
ACE: angiotensin converting enzyme; ARB: angiotensin II receptor blocker; BICD: biventricular implantable cardioverter defibrillator, BMI: body mass index; ICD: implantable cardioverter defibrillator; RAASi: renin-angiotensin-aldosterone system inhibitor; SE: standard error.<br>*SE calculation based on a beta distribution			

2.2. Initial distribution of disease status

The distribution of patients by CKD and HF status at the start of treatment was informed by Italian sources using the values presented in **Table 3.**

Table 3. Disease severity at start of treatment in the Italian population.

	Mean	SE	Source
**Starting health state distributions**			
Proportion with HF*	0.360	-	Provenzano et al.[4]
Proportion CKD Stage 3	0.430	0.0212	Provenzano et al.[4]
Proportion CKD Stage 4	0.445	0.0213	
Proportion CKD Stage 5	0.125	0.0142	
**Patient characteristics**			
Age (years)	66.4	0.57	Provenzano et al.[4]
Proportion female	0.41	0.02	
CKD: chronic kidney disease; HF: heart failure; SE: standard error<br>*proportion with cardiovascular disease, including heart failure, myocardial infarction, peripheral vascular disease and stroke			

References

1. Santoro A, Perrone V, Giacomini E, Sangiorgi D, Alessandrini D, Degli Esposti L (2022) Association between hyperkalemia, RAASi non-adherence and outcomes in chronic kidney disease. J Nephrol;35(2):463-72. doi:10.1007/s40620-021-01070-6.
2. Volterrani M, Perrone V, Sangiorgi D, Giacomini E, Iellamo F, Degli Esposti L (2020) Effects of hyperkalaemia and non-adherence to renin-angiotensin-aldosterone system inhibitor therapy in patients with heart failure in Italy: a propensity-matched study. Eur J Heart Fail;22(11):2049-55. doi:10.1002/ejhf.2024.
3. Maggioni AP, Dondi L, Andreotti F, Calabria S, Iacoviello M, Gorini M, Gonzini L, et al. (2021) Prevalence, clinical impact and costs of hyperkalaemia: Special focus on heart failure. Eur J Clin Invest;51(8):e13551. doi:10.1111/eci.13551.
4. Provenzano M, Minutolo R, Chiodini P, Bellizzi V, Nappi F, Russo D, Borrelli S, et al. (2018) Competing-Risk Analysis of Death and End Stage Kidney Disease by Hyperkalaemia Status in Non-Dialysis Chronic Kidney Disease Patients Receiving Stable Nephrology Care. J Clin Med;7(12). doi:10.3390/jcm7120499.
5. Provenzano M, De Francesco M, Iannazzo S, Garofalo C, Andreucci M, Genualdo R, Borrelli S, et al. (2020) Cost-analysis of persistent hyperkalaemia in non-dialysis chronic kidney disease patients under nephrology care in Italy. Int J Clin Pract;74(5):e13475. doi:10.1111/ijcp.13475.
6. Levy WC, Mozaffarian D, Linker DT, Sutradhar SC, Anker SD, Cropp AB, Anand I, et al. (2006) The Seattle Heart Failure Model prediction of survival in heart failure. Circulation;113(11):1424-33.
