# Supplementary material for: Hyperkalemia in chronic kidney disease patients with and without heart failure: an Italian economic modelling study
Source: Cost Eff Resour Alloc. 2024 May 21;22:42. doi: 10.1186/s12962-024-00547-y (PMC11106859; doi:10.1186/s12962-024-00547-y)
Supplement: Supplementary file 6 — Additional file 6: Subgroup analysis. Provides details of cumulative clinical events in patients with CKD and HK versus CKD alone [file 12962_2024_547_MOESM6_ESM.pdf]

Additional file 6

Cumulative number of clinical events in patients with CKD and HK versus CKD alone.

Table 1: Cumulative number of clinical events in patients with CKD

	CKD and HF				CKD only			
**Cumulative events/patient**	**Treatment**	**Control**	**Incremental**	**NNT**	**Treatment**	**Control**	**Incremental**	**NNT**
Time on treatment (months)	9.469	0.000	9.469	N/A	11.076	0.000	11.076	N/A
Treatment repeat	0.341	0.000	0.341	N/A	0.482	0.000	0.482	N/A
Treatment discontinuation	1.341	0.000	1.341	N/A	1.482	0.000	1.482	N/A
HK (K+ > 5.5 to ≤ 6)	1.534	1.761	-0.228	4.4	1.711	1.932	-0.221	4.5
HK (K+ > 6)	0.370	0.423	-0.053	19.0	0.419	0.469	-0.051	19.7
MACE	2.569	2.640	-0.071	14.1	2.979	2.965	0.014	N/A
Hospitalisation	3.202	3.199	0.003	N/A	2.537	2.465	0.073	N/A
RAASi discontinuation	1.984	2.218	-0.234	4.3	2.673	2.875	-0.201	5.0
RAASi down-titrate	1.057	1.096	-0.039	26	1.393	1.377	0.016	N/A
RAASi up-titrate / restart	1.552	1.739	-0.187	5	2.213	2.390	-0.177	6
Dialysis	0.424	0.422	0.001	N/A	0.585	0.570	0.015	N/A
Transplant	0.211	0.210	0.001	N/A	0.297	0.290	0.007	N/A
Dialysis complications	0.078	0.078	0.000	N/A	0.110	0.107	0.003	N/A
Mortality	1.000	1.000	0.000	N/A	1.000	1.000	0.000	N/A

CKD: chronic kidney disease; HF: heart failure; MACE: Major advance cardiac event; NNT: Number needed to treat; RAASi: renin–angiotensin–aldosterone system inhibitor
