# Supplementary figures and images for: Hyperkalemia in chronic kidney disease patients with and without heart failure: an Italian economic modelling study
Source: Cost Eff Resour Alloc. 2024 May 21;22:42. doi: 10.1186/s12962-024-00547-y (PMC11106859; doi:10.1186/s12962-024-00547-y)

**Additional file 7**

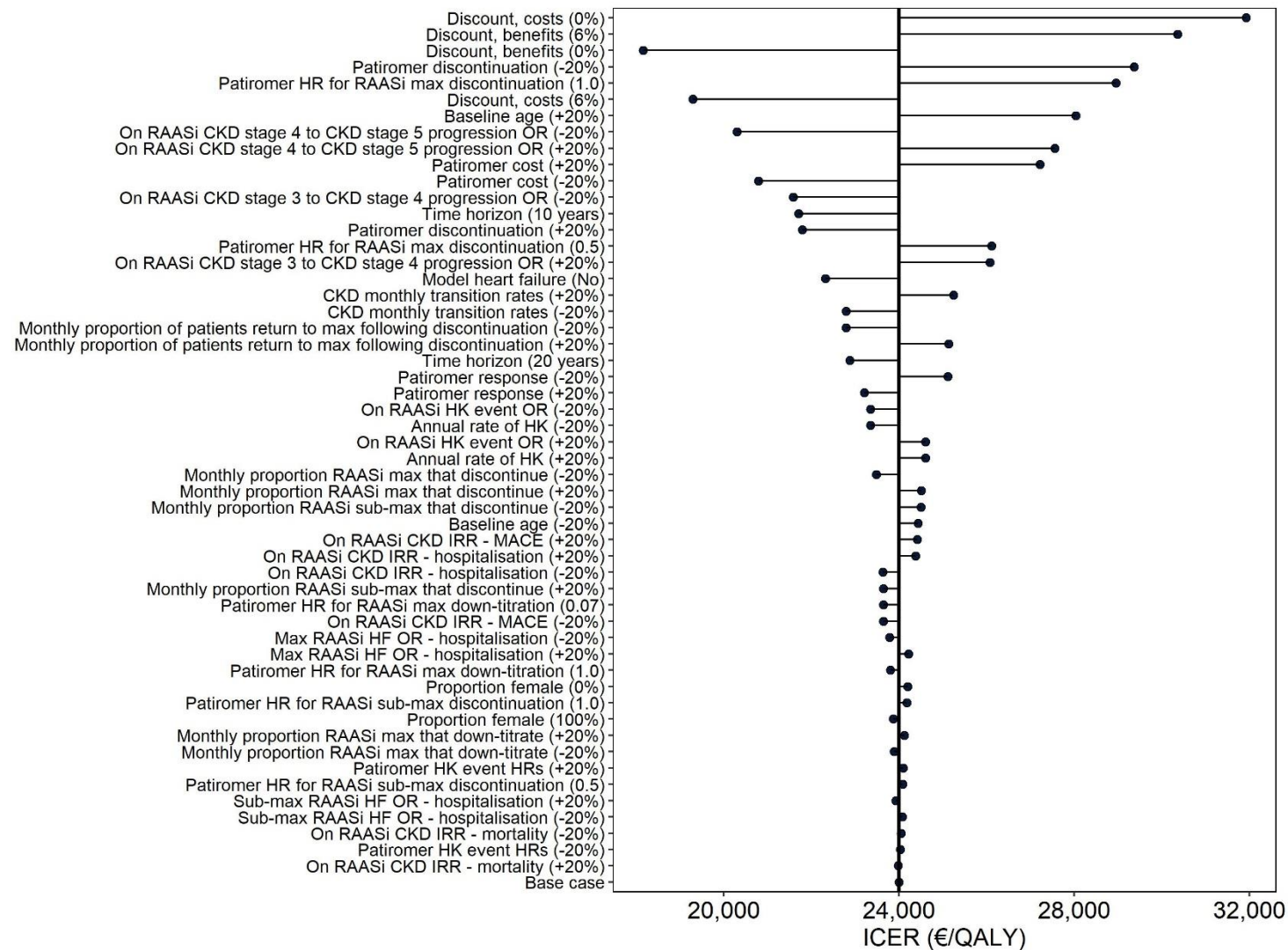

**Figure 1:** Results of deterministic sensitivity analysis.

Supplement: Supplementary file 7 — Additional file 7: Sensitivity analysis tornado. Provides results of deterministic sensitivity analysis [file 12962_2024_547_MOESM7_ESM.pdf]
